# The fractal organization of ultradian rhythms in avian behavior

**DOI:** 10.1038/s41598-017-00743-2

**Published:** 2017-04-06

**Authors:** Diego A. Guzmán, Ana G. Flesia, Miguel A. Aon, Stefania Pellegrini, Raúl H. Marin, Jackelyn M. Kembro

**Affiliations:** 1 Instituto de Investigaciones Biológicas y Tecnológicas (IIByT-CONICET); Instituto de Ciencia y Tecnología de los Alimentos, Cátedra de Química Biológica, Facultad de Ciencias Exactas, Físicas y Naturales, Universidad Nacional de Córdoba, 1611 Vélez Sarsfield, X5016GCA Córdoba, Córdoba Argentina; 2 Department of Animal Science, Aarhus University, 20 Blichers Allé, Post Box 50, DK-8830 Tjele, Denmark; 3 Centro de Investigaciones y Estudios de Matemática (CIEM - CONICET); Facultad de Matemática, Astronomía y Física FAMAF, Universidad Nacional de Córdoba, Ing. Medina Allende s/n Ciudad Universitaria, Córdoba, CP X5000HUA Argentina; 4 Johns Hopkins University School of Medicine, 720 Rutland Avenue, Ross Bldg. 1059, Baltimore, MD 21205 USA; 5 National Institute on Aging/NIH, BRC, 251 Bayview Blvd., Baltimore, MD 21224 USA

## Abstract

Living systems exhibit non-randomly organized biochemical, physiological, and behavioral processes that follow distinctive patterns. In particular, animal behavior displays both fractal dynamics and periodic rhythms yet the relationship between these two dynamic regimens remain unexplored. Herein we studied locomotor time series of visually isolated Japanese quails sampled every 0.5 s during 6.5 days (>10^6^ data points). These high-resolution, week-long, time series enabled simultaneous evaluation of ultradian rhythms as well as fractal organization according to six different analytical methods that included Power Spectrum, Enright, Empirical Mode Decomposition, Wavelet, and Detrended Fluctuation analyses. Time series analyses showed that all birds exhibit circadian rhythms. Although interindividual differences were detected, animals presented ultradian behavioral rhythms of 12, 8, 6, 4.8, 4 h and/or lower and, irrespective of visual isolation, synchronization between these ultradian rhythms was observed. Moreover, all birds presented similar overall fractal dynamics (for scales ∼30 s to >4.4 h). This is the first demonstration that avian behavior presents fractal organization that predominates at shorter time scales and coexists with synchronized ultradian rhythms. This chronobiological pattern is advantageous for keeping the organism’s endogenous rhythms in phase with internal and environmental periodicities, notably the feeding, light-dark and sleep-wake cycles.

## Introduction

The temporal organization of biochemical and physiological processes involved in animal behavior such as hormonal secretion, sleep, locomotion, has evolved periodicities that match the external world, and endogenous spontaneous patterns that can be complex and non-periodic^1–3^. In living systems these developed patterns provide internal coordination to maintain spatial and temporal organization^4^. For example, the timing of the circadian clock helps to anticipate and respond to environmental changes while adjusting accordingly^5^. However, biological timing of processes diverse in nature (chemical, electrical, mechanical, hormonal) is complex because they are nonlinearly interrelated, and their temporal organization occurs on many time scales simultaneously^6, 7^. Non-linearity of functional activities is foundational of the homeodynamic (rather than homeostatic) nature of the living state^8, 9^ and leads to the emergence of periodic dynamics comprising oscillatory rhythms and clock-timed output controls^7^. Unlike oscillations and rhythms, circadian or ultradian clocks have a temperature-compensating mechanism that makes them independent from this factor, as one would expect from a reliable clock^10, 11^. Oscillators, rhythms and clocks span many time domains from milliseconds to years^7^. Indeed, biological timekeeping requires more than circadian (24 h) organization. Coordination on the ultradian (<24 h) domain (i.e. faster time scales where clocks cycle many times in a day) is essential^6, 7, 12^. In contrast with circadian, ultradian rhythms exhibit greater inter-individual variability^12^ and might not always be detected in subjects from the same species, age, and sex. For example, only 52% of male and 42% of females exposed to a normal 15 L:9D cycle in 10 week old Siberian hamsters exhibited dark-phase ultradian locomotor rhythms, with females showing longer periods than males (∼3 *vs*. ∼2 h)^13^. However, synchronization of ultradian rhythms in animal groups have been reported^14–16^.

In addition to rhythmic behavior, a large body of evidence indicates that some physiological (e.g. heart rate) and behavioral (e.g. locomotor activity, swimming patterns, social behavior) processes exhibit robust scale-invariant fractal patterns and long-range temporal correlations^3, 17–20^. Fractals can be of geometric, statistical and dynamical nature^17, 21^. They are composed of parts that at different magnification scales resemble the whole, i.e. they are self-similar. The self-similarity trait of fractals underlies their scale-invariance that, when applied to time series, renders fractal temporal fluctuations across multiple time scales^22, 23^. The scaling and self-similarity traits of fractals are fundamentally important for physiology and biochemistry because they reveal the intrinsic interdependence among the different functional levels of organization exhibited by living systems. In this context, fractal behavior represents an emergent property from underlying multi-scale physiological processes and their environmental interactions^24^. Specifically, the fractal dynamics of animal locomotion has been proposed to be regulated by both the central and intraspinal components of the nervous system, involving feedback loops, a central pattern generator^25–27^, psychophysical control^28^, and intrinsic activity control mechanisms^29^.

Locomotion is a convenient, non-intrusive, way to measure circadian and ultradian rhythms^30^. In addition, locomotion patterns from numerous vertebrate species exhibit fractal dynamics^18, 19, 24^. Mathematically speaking, fractals in their infinite, but reducible complexity can be considered opposites of smooth oscillatory rhythms. However, unknown is whether ultradian rhythms and the fractal dynamics observed in animal locomotion across vertebrate taxa can coexist. We also ask whether behavioral dynamic patterns will resemble in all animals studied, or whether they will show inter-individual variability. To address these questions we herein analyze locomotion of individually housed Japanese quails in a home-cage environment, visually isolated from conspecifics, at high sampling rate (every 0.5 s) recorded continuously over a 6.5 day period. The high temporal resolution series of avian motion obtained (1.07 × 10^6^ data points) enabled detection of circadian and ultradian rhythms along with fractal dynamics over a broad range of temporal scales. In this experimental setup quails do not fly but walk, thus allowing comparison with previous locomotion studies in mammals.

## Results

### Characterization of circadian and ultradian rhythms in time series of quail locomotor activity

**Figure 1:**
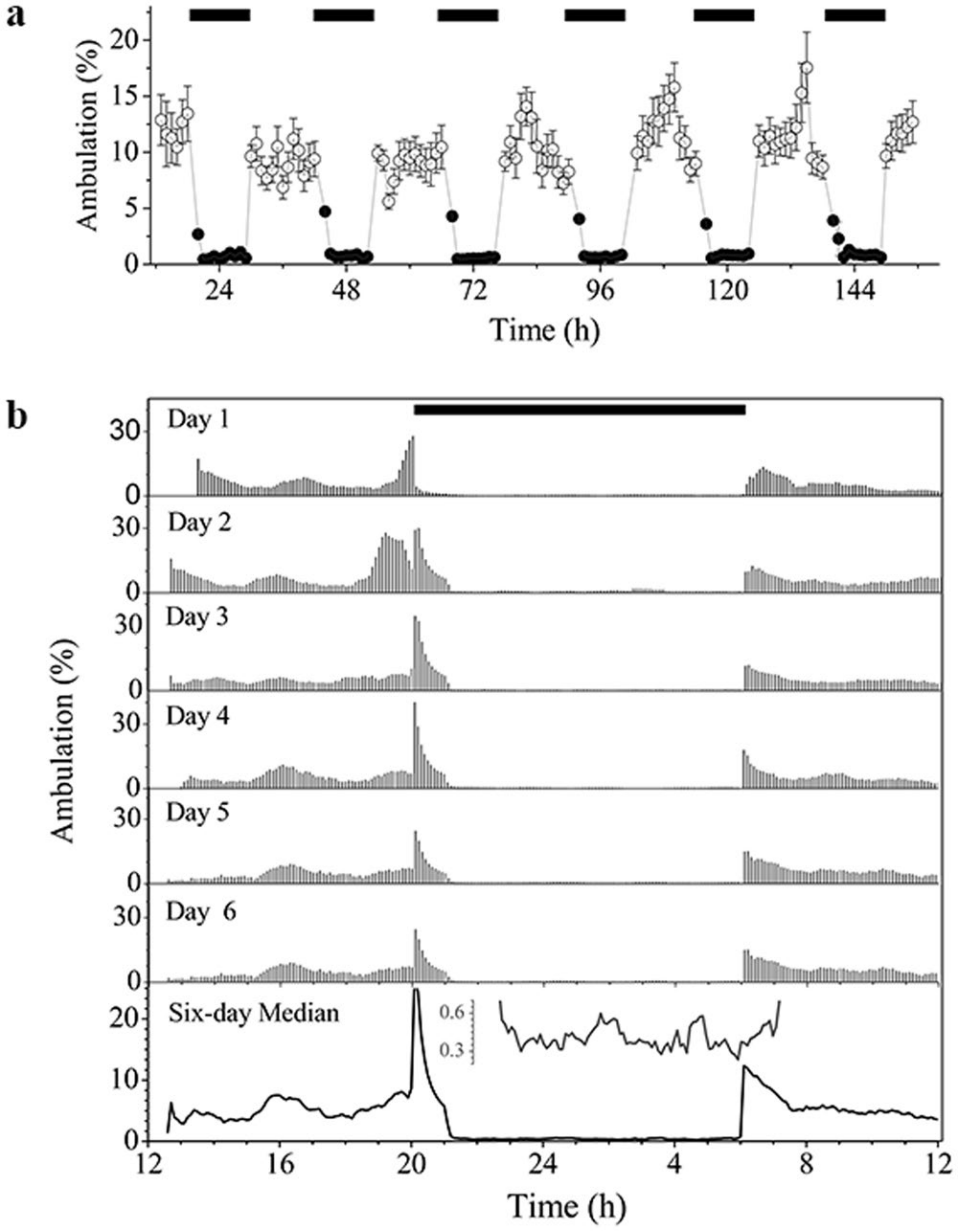
Locomotor activity of female Japanese quail in a home-cage environment. (**a**) Hourly mean values of the percent of time spent ambulating of the 24 quails tested (Mean ± SEM). (**b**) Representative actogram of an animal with both ultradian and circadian rhythms (see analysis of this time series in Figs 2 and 3). Actogram was constructed using 6-min bins, and the percentage of time spent ambulating in each 6 min bin ranged from 0 to 45%. Black bar represents the dark period. Last panel represents the median actogram estimated by calculating the median percentage of time spent ambulating in each 6 min bin over the 6 day period. The inset displays a magnification of the nighttime period (20–34 h). Peaks in median actogram provide visual evidence of underlying ultradian (<24 h) rhythms, both during daytime and nighttime periods.

Quails are diurnal birds that show predominantly locomotor activity during daylight hours. After dark, locomotion continues during the first hour dropping to a minimum thereafter (Fig. 1a). Inspection of actograms of quail locomotion revealed both circadian and ultradian rhythms (Fig. 1b). Properly magnified, the nighttime period also shows rhythmic activity in all animals studied (Fig. 1b, inset bottom panel).

**Figure 2:**
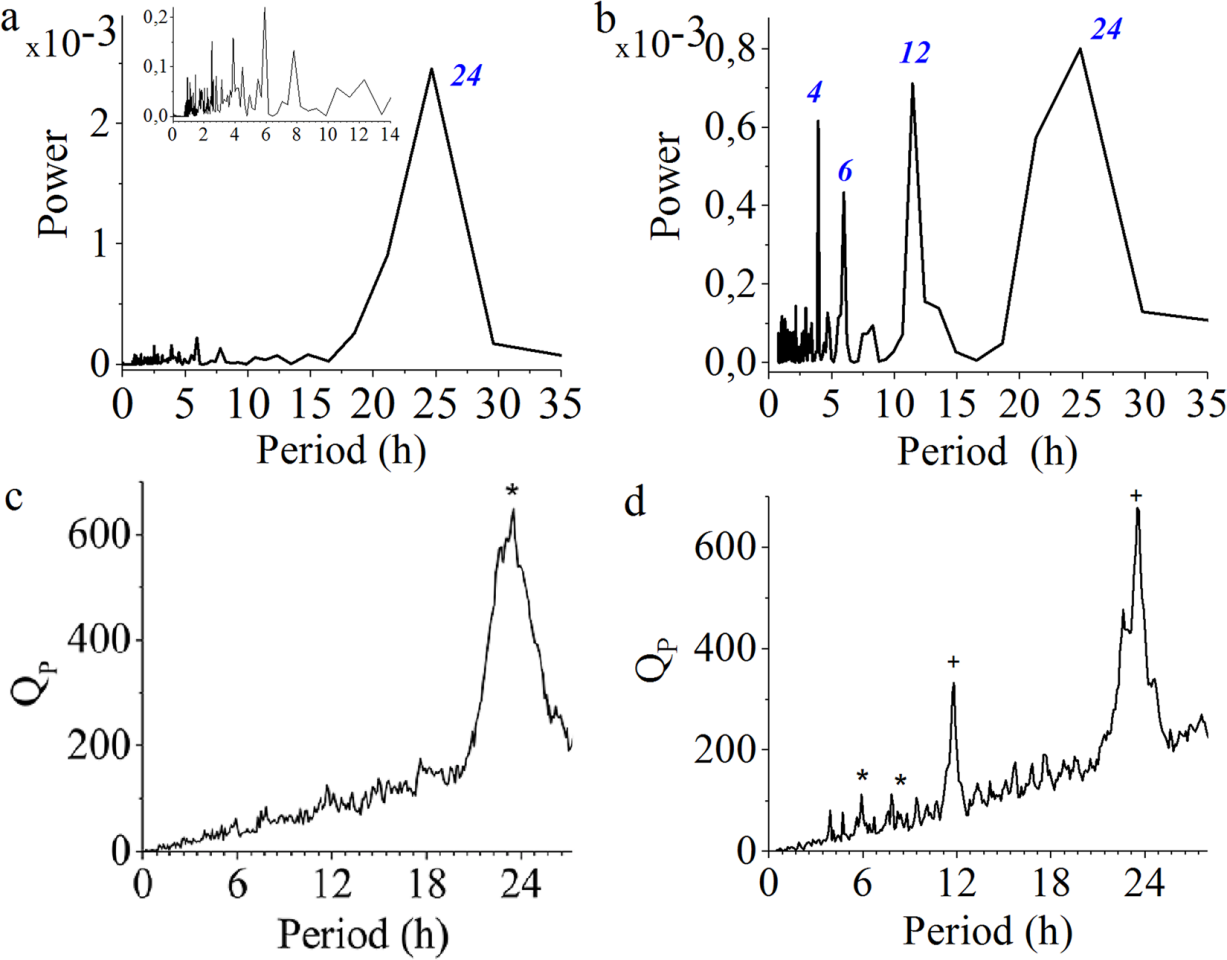
Analysis of rhythms in locomotion time series. Analytical examples of (**a,b**) power spectrum, and (**c,d**) Enright’s method corresponding to two time series of locomotor activity of female Japanese quail in a home-cage environment. (**a,c**) The time series analyzed clearly present circadian rhythms but do not show evidence of ultradian rhythms. Blue italic numbers represent the period in hours of the peaks. (**b,d**) Analysis of the same time series depicted in Fig. 1b. Of note is that the two methods detect both circadian and ultradian rhythms in this time series. Complete high-resolution time series (10^6^ data points) were used for power spectrum. To reduce the impact of noise in the analysis, we applied the Enright’s method to actograms (6 min bins). *Statistically significant peaks (P < 0.001). ^+^These peaks are multiples of 6 and therefore the algorithm does not estimate significance.

**Figure 3:**
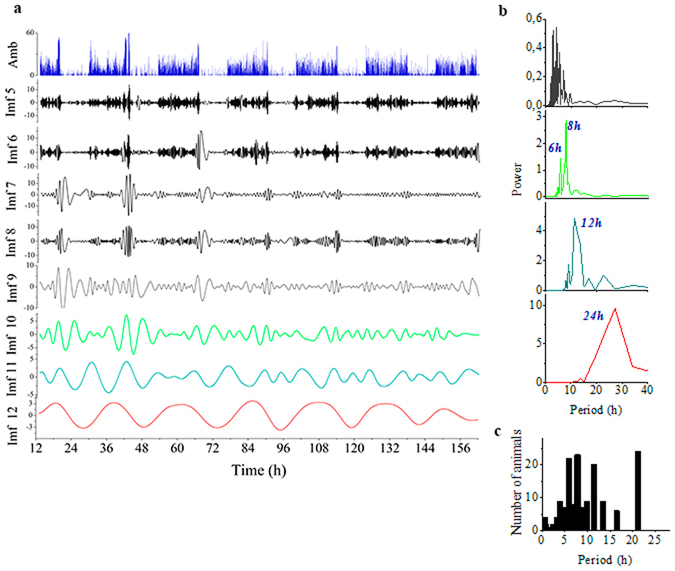
Empirical Mode Decomposition (EMD) of time series in ambulatory activity. (**a**) EMD of the actogram constructed using a 30 s time window (top panel in blue). For visualization, of the 15 Intrinsic Mode Functions (IMFs) found only 9 are shown (IMFs 5–12). Note the smooth oscillations observed in all IMFs. The Power Spectrum Analysis (PSA) of IMFs 9–12 are depicted in (**b**). For EMD a 30 sec window was selected as a tradeoff between the need for a large number of data points for PSA, and the advantage of smoothing the time series by pooling data in larger time windows. (**c**) A histogram of the principle peaks in PSA of IMFs detected in all animals is shown. Note that circadian and ultradian rhythms were observed in all animals, namely with periods of 24 h, 12 h, 8 h, 6 h and 4 h. Principal peaks was defined as the largest 10 peaks that appear at least once in the PSA of a subgroup of 4 IMFs (equivalent to the 4 selected in “c”), where the height of the peak was larger than 30% of the power of the highest peak of the corresponding PSA.

The characterization of the oscillatory rhythmic behavior was performed with Power Spectra, Enright’s, Empirical Mode Decomposition, and Wavelet analyses. All four methods detected circadian rhythms (∼24 h) in all animals (Figs 2 and 3). Power spectra from all animals exhibit a significant peak at 24 h (Fig. 2a,b) and clear evidence of at least one significant ultradian rhythm (Fig. 2b) detected in 21 out of 24 animals; in the three birds where the ultradian rhythms were not apparent, proper scale magnification enabled their visualization somewhat obscured by the strong power of the circadian rhythm in these animals (Fig. 2a). The Enright’s method detected circadian rhythms but the output in the ultradian domain was noisy (Fig. 2c,d). Nineteen out of twenty four animals exhibited at least one significant ultradian rhythm according to the Enright’s method. Specifically, 41%, 33% and 18% of the animals presented significant 12 h, 8 h and 6 h ultradian rhythms, respectively.

Empirical Mode Decomposition (EMD, a data-driven method that detects the strongest functions underlying a given signal) decomposed each locomotor time series in more than 14 Intrinsic Mode Functions (IMFs). An example is shown in Fig. 3, where EMD revealed 15 IMFs, of which eight (IMFs 5–12) are depicted (Fig. 3a). Noteworthy, is the smooth oscillatory nature of these IMFs, of which four correspond to 24 h, 12 h, 6 h, and 4 h rhythms according to Fourier spectra analysis (Fig. 3b). Rhythms of shorter periods can also be observed in the remaining IMFs which progressively include more than one frequency (i.e. mode mixing). A histogram with the predominant peaks corresponding to the circadian and ultradian rhythms of 24 h, 12 h, 8 h, 6 h and 4 h found in the Power Spectrum of IMFs of all 24 quails is depicted in Fig. 3c. Overall, EMD analysis provides clear evidence of the presence of ultradian rhythms as well as the smooth oscillatory nature of both circadian and ultradian rhythms.

**Figure 4:**
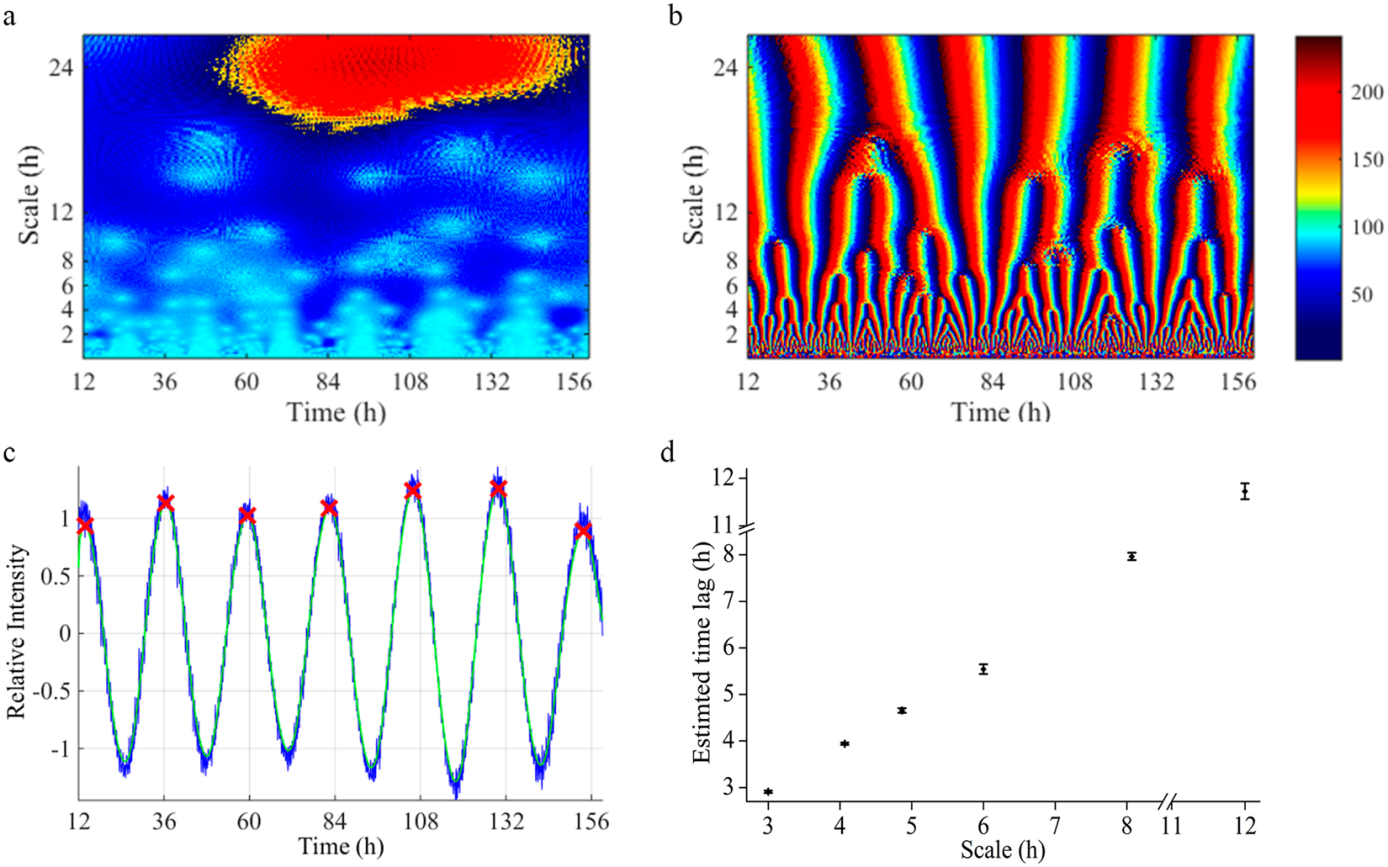
Wavelet analysis of ambulatory activity time series. Amplitude plots of complex valued Morlet transform: (**a**) Modulus, and (**b**) phase angle. The *x*-axis represents time (6.5 days) and the *y*-axis indicates the scale of the wavelet used (from 6 min to 26 h). The wavelet decomposition reveals an overall self-similar fractal structure particularly noticeable at low scales, while the periodic behavior of the series is more clearly depicted at larger scales. Similar plots were obtained for all animals studied. (**c**) An example of estimation of time lag between peaks in the relative intensity of the real wavelet coefficients at the 24 h scale is shown. Cross symbols in red (x) indicate peak values, while the distance between peaks is the lag time. For examples of lag time estimation for smaller scales see Supplementary Fig. 5. (**d**) Mean ± SEM of the lag time between peaks is represented for all 24 animals evaluated as a function of the time scale. Notice that the lag time corresponds with the time scale evaluated, hence providing analytical confirmation of the presence of these rhythms in the locomotor activity time series. Wavelet analysis was performed on actograms with 6 min bins in order to reduce noise (see Supplementary Fig. 3 for comparison with wavelet analysis performed on complete time series).

Wavelet analysis was able to not only detect ultradian rhythms of 12 h, 8 h, 6 h, 4.8 h, 4 h, 3 h, 2.4 h, 2 h periods, and even lower, but also to unveil their localization in the time series from all birds. The successive branching of the period from these oscillatory rhythms exhibits a fractal pattern. Figure 4 depicts the amplitude of seven peaks corresponding to the wavelet coefficients (red blots) in phase angle with 24 h circadian rhythmicity (Fig. 4b). This pattern then ramifies, resulting in 13 amplitude peaks in the 12 h scale (2 peaks a day), that corresponds to the 12 h ultradian rhythm. This ramification process then repeats at successively shorter temporal scales, e.g. 19, 25, 30, and 38 peaks in amplitude are observed at 8, 6, 4.8, and 4 h scales, respectively (Supplementary Fig. 4). Strikingly, the successive bifurcation at different temporal scales leads to a manifestly fractal pattern at shorter time periods (Fig. 4 bottom panel). Overall, wavelet analysis reveals a complex self-similar fractal pattern in the quail’s locomotion resulting from the recursive doubling of a main branch into two branches as in pulmonary airways and blood vessels.

**Figure 5:**
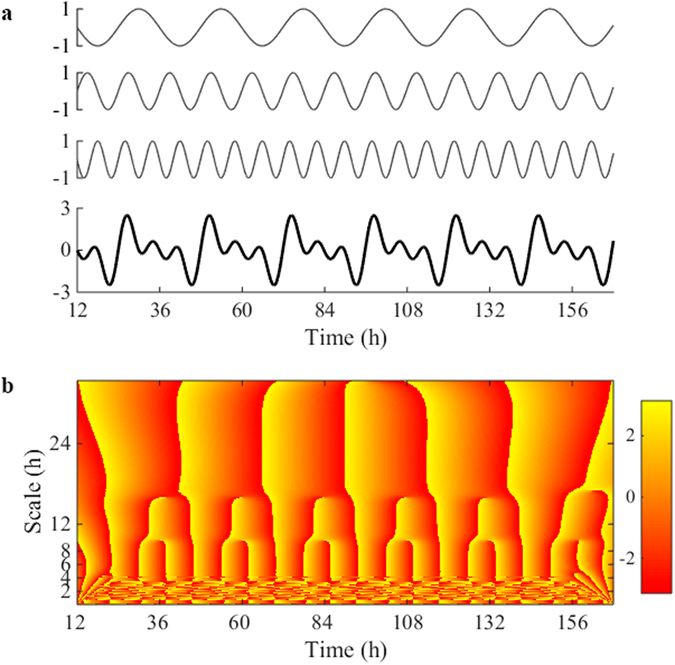
Example of wavelet analysis of ultradian rhythms using a synthetic time series. (**a**) Example time series constructed as the sum of three sine waves of the same amplitude but different periods corresponding to 24 h, 12 h and 8 h (from top to bottom panel), considering a duration of 6.5 days and a 0.5 s sampling rate. (**b**) Amplitude plots of phase angle of the wavelet Morlet transform. The *x*-axis represents time (6.5 days) and the *y*-axis indicates the scale of the wavelet used (from 6 min to 26 h). Notice the similarities with Fig. 4 in the branching pattern for time scales ≥ 8 h.

**Figure 6:**
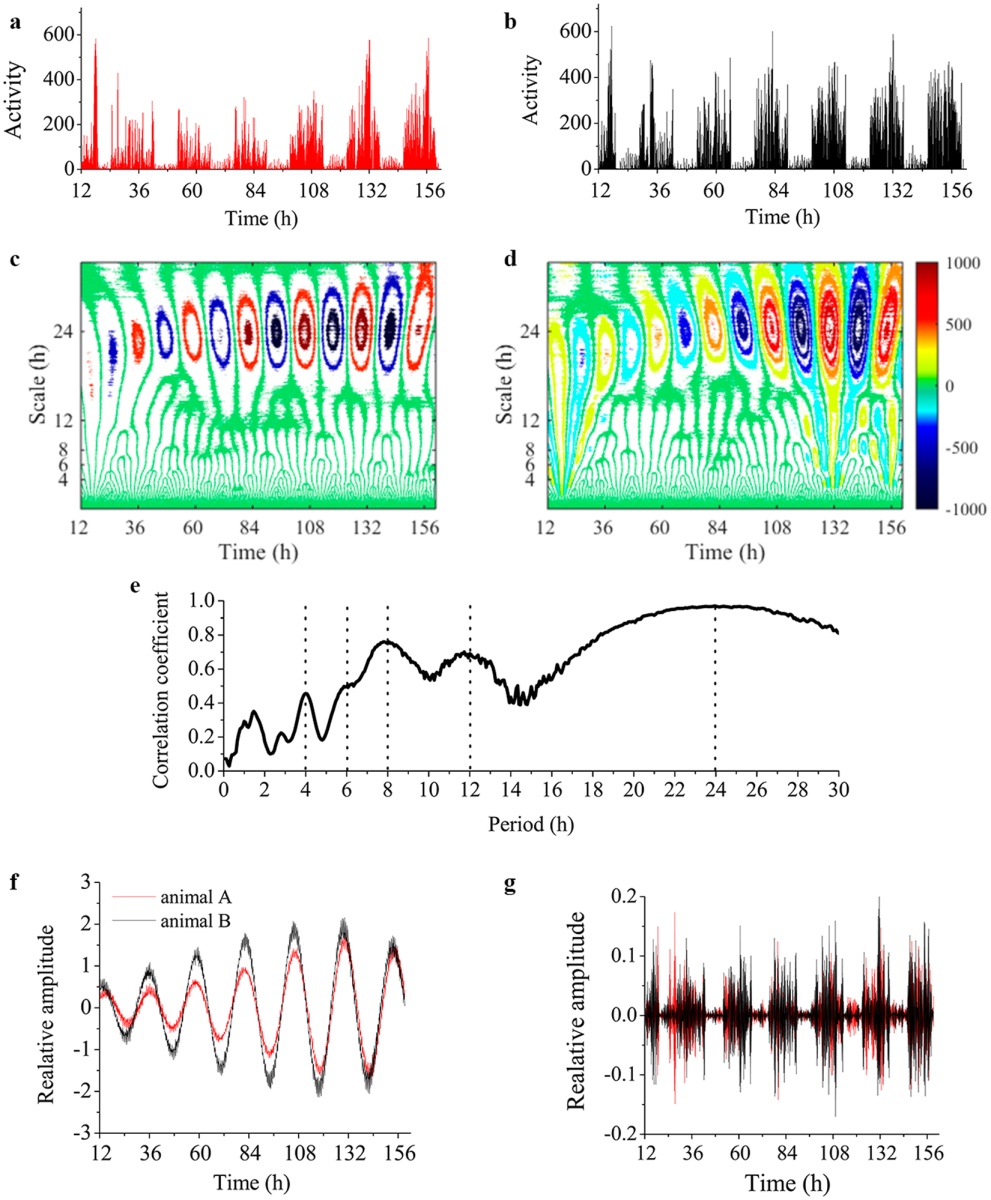
Synchronicity of ultradian rhythms. (**a,b**) Comparative actograms of locomotion time series (6 min bins) from two female Japanese quails. (**c,d**) Contour plot of the real part of the wavelet coefficients estimated with a complete Morlet wavelet for time series “a” and “b”. The *x*-axis represents time (6.5 days) and the *y*-axis indicates the scale of the wavelet used (from 6 min to 26 h). Yellow and red indicates higher values while blue indicates minimal amplitude values. (**e**) Representation of the diagonal correlation coefficients obtained from comparing the real component of the wavelet coefficients for the same two animals at each time scale using the Spearman correlation coefficient. Note that these correlation coefficients denote the time interval where there is effective correlation and the temporal scales at which the maximum value of the coefficients occurs. Depicted is an example of the amplitude of the wavelet coefficients at 24 h (**f**) (maximal correlation), and 0.3 h (**g**) (minimal correlation) for time series “a” and “b”. Average correlation coefficients at each time scale estimated from all pair-wise comparison between all 12 animals from the same experiment is represented in Supplementary Fig. 6.

To further ascertain the reliability of the wavelet analysis employed, in Fig. 5 we show an example corresponding to a synthetic time series constructed as the sum of rhythms with different periods (24 h, 12 h and 8 h) which also results in a branching pattern. Of note is that the peaks in the phase angle occur at the expected scale coinciding with peak values in the signal time series, and that the branching pattern disappears for temporal scales <8 h period. This same phenomenon is observed in locomotion data, e.g. compare panels *a* and *c* in Fig. 6, where peak day activity coincides with peak amplitude (red blot) in the real coefficients of the Morlet transform (*Re*(*cwt*)), while minimum nighttime activity matches with low amplitude values (blue blots).

Amplitude plots of the real part of the wavelet coefficients (*Re*(*cwt*)) displayed similar patterns (Fig. 6c,d) as those shown by the phase angle (Fig. 4) in all 24 animals. Further confirmation of this important result was obtained by quantification of the lag time between peaks in the relative intensity of the coefficients at different time scales (Fig. 6e,f; Supplementary Fig. 5). The underlying rationale of this procedure is that if a certain period is present in a rhythm then the lag time between peaks should approximate the value of the time scale at which the lag was calculated (Fig. 6f)^30^.

We applied the Pering’s technique to determine whether the appearance of peaks in the amplitude corresponding to the Re(cwt) was synchronous between different, visually isolated, animals. Firstly, the continuous wavelet transform (cwt) followed by the Spearman’s rank correlation coefficient were calculated; secondly, we generated a graphical representation of links between periodicities present in the two signals (Fig. 6, and Supplementary Fig. 6)^31^. We found that, even for periods ≤4 h, synchronization between different animals from both experimental groups could be detected (Supplementary Fig. 6), as can be judged from peak amplitude localization with respect to the Re(cwt) and the corresponding temporal scale of the ultradian rhythm. An example of amplitude variations according to Re(cwt) at 24 h (maximal correlation, Fig. 6f) and 0.3 h (minimal correlation, Fig. 6g) is shown for two animals. Evident from these plots is that a positive value of the correlation coefficient at a given temporal scale (24 h) indicates not only that the two animals compared present a peak in their rhythmic activity at that scale, but also specifies their degree of synchronization with respect to the time of the day at which the rhythm appears. For example, the 12 h ultradian rhythm shows peak amplitude around 2 PM and 2 AM whereas the 6 h rhythm shows peaks approximately at 7:00 AM, 1:00 PM, 7:00 PM, and 1:00 AM (Supplementary Fig. 6).

To rule out potential rhythm entrainment by daily cage maintenance activity, we ran an independent control experiment where completely undisturbed quails’ locomotor activity was monitored during 5.5 days. Equivalent rhythms with periods of 24 h, 12 h, 8 h, 6 h and lower were revealed by power spectrum, EMD and wavelet analyses in control undisturbed (Supplementary Figs 7–9) in comparison to maintenance-disturbed (Figs 2–4) birds. These results are in agreement with the idea that the ultradian rhythms observed are of endogenous origin rather than the result of entrainment by exogenous factors other than light-dark, sleep-wake and feeding cycles.

### Characterization of fractal dynamics

**Figure 7:**
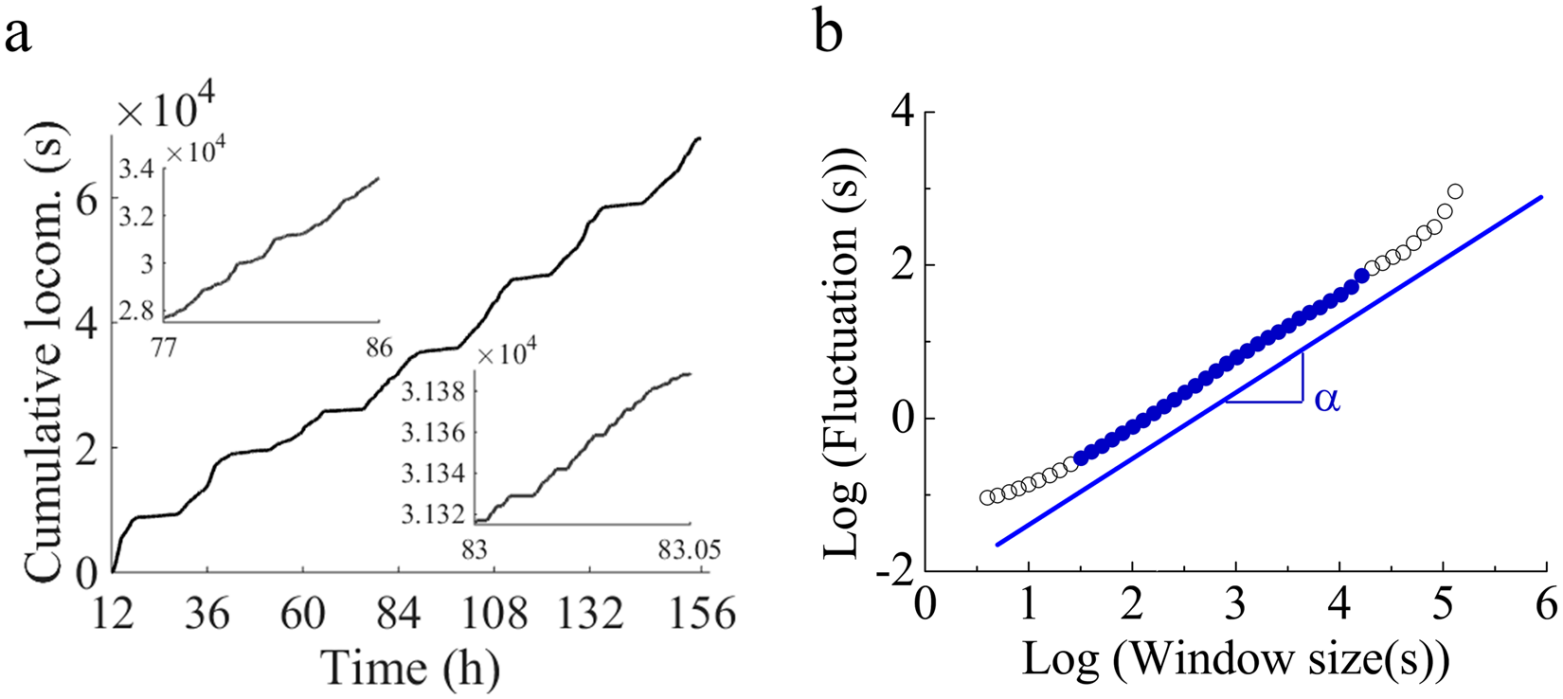
Scale-invariance and long-range correlations in locomotor activity. (**a**) Cumulative locomotion activity of a quail in a home-cage environment. Locomotion was monitored at 0.5 s interval (x_i_), if the bird was ambulating, x_i_ = 1, and if immobile x_i_ = 0. (**b**) DFA of order 3 (DFA3) of the time series shown in panel a. The double log plot of fluctuation *vs*. window size is linear (blue line) in the region shown with blue closed circles (●) with slope “α“, a metric of self-similarity. Of note is that for larger scales there is no evidence of loss of fractal behavior although the estimation of α in this region is unreliable (Supplementary Fig. 11). DFA of order 3 was selected given that it was the lowest order of DFA that eliminated trends in all locomotor time series (Supplementary Fig. 10).

The fractal dynamics determined by wavelet analysis was further confirmed and characterized by Detrended Fluctuation Analysis (DFA) and frequency histograms of immobility events. DFA showed that 83% of birds exhibit long-range correlations in the locomotion time series for scales ranging from 30 s to 23 h, while the remaining 17% present a slight deviation from scaling for large time scales (>4.4 h: Supplementary Fig. 10). Quail’s locomotion (Fig. 7a) presented a value of the self-similarity parameter α ranging from 0.738 to 0.907 (mean = 0.850 ± 0.008; r^2^ > 0.998) for temporal scales spanning from ∼30 s to >4.4 h. This α-value indicates that the temporal dynamics of locomotion in all birds is fractal, presenting self-similarity and long-range correlations (i.e. present behavior depends on past behavior^32^) for temporal scales ranging from ∼30 s to >4.4 h (Fig. 7b). Applying an appropriate filter to the time series showed that DFA eliminates the effect of the underlying circadian rhythm on the long-term temporal correlations (Supplementary Fig. 11a). Additionally, we determined that α obtained from filtered time series was similar and highly correlated with the original unfiltered data (Supplementary Fig. 11b). Similar results were obtained with an independent approach involving power spectral analysis (Supplementary Fig. 17).

**Figure 8:**
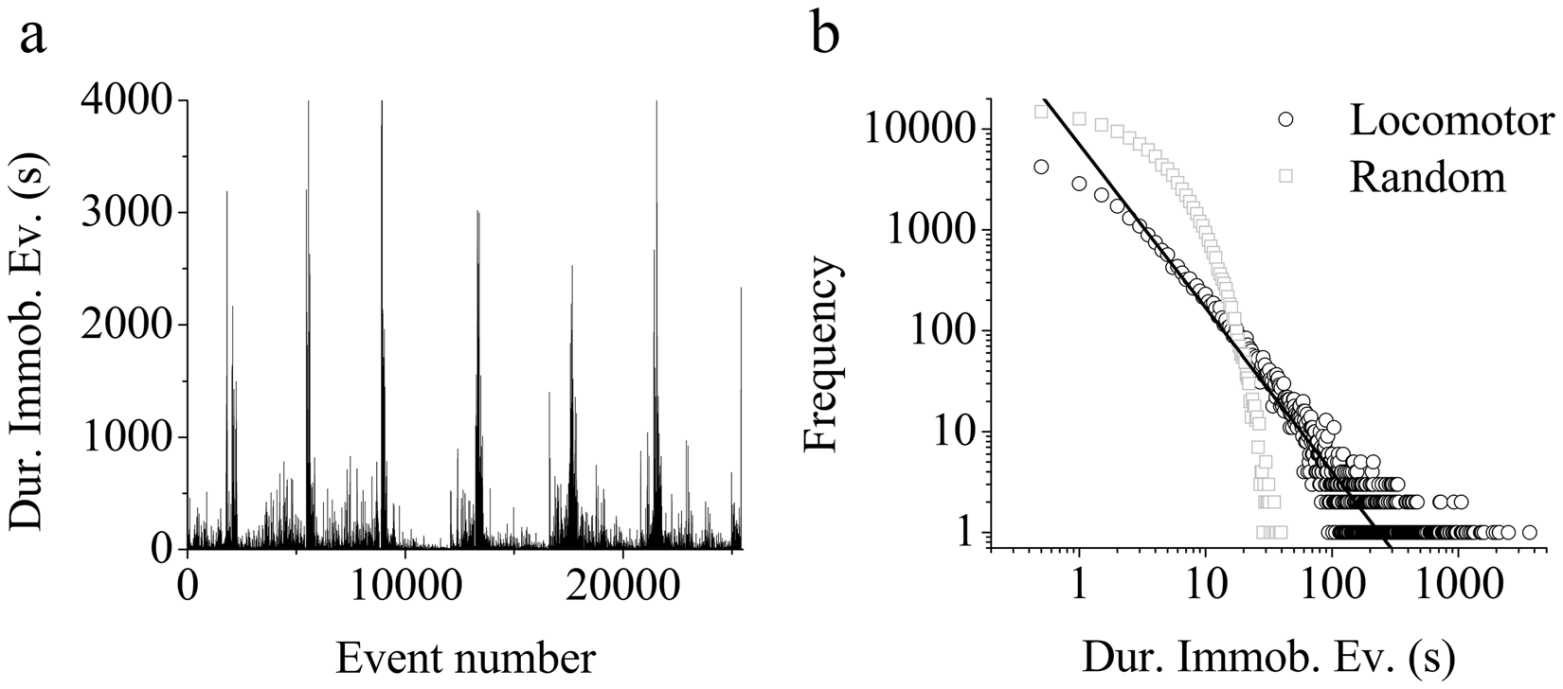
Power-law (fractal) distribution of the duration of immobility events. (**a**) Immobility event duration as a function of the event number for the same animal time series represented in Fig. 6. (**b**) Frequency distribution of the immobility events computed from the 6.5 days of continuous recording (black open circles). Solid line corresponds to the linear fit in the log-log plot, y = −1.62x + 3.85 (r^2^ = 0.91), estimated for event sizes from 1 to 250 s (see Supplementary Figure for 13 for details on criteria for selecting scaling region), indicating fractal behavior according to an inverse power law. The same time series was randomized and then reanalyzed (grey open squares) for comparison. Random data fits an exponential distribution (r^2^ = 0.86). Complete high-resolution time series (10^6^ data points) were used for event duration estimation.

The quail’s activity showed many immobility events of long duration (Fig. 8a). The distribution of the frequency of immobility events obeys an inverse power law with a pronounced non-Gaussian tail (Fig. 8b), suggesting a fractal distribution. The long tail of the power law indicates that immobility during long periods of time is less frequent while the opposite occurs with short periods of immobility. Likewise, a steeper slope S of the frequency distribution on a double log plot (Fig. 8b) indicates the presence of a greater number of events with short rather than long duration. We determined S-values of −1.52 ± 0.02 (range −1.32 and −1.64; r^2^ > 0.89) for durations between 1 s and 4 min. Whether the behavior observed is non-random was ascertained by showing that the randomized original time series present an exponential rather than a power law distribution (Fig. 8b).

## Discussion

The main contribution of the present work is to show that the locomotor activity of quails exhibits a temporal architecture obeying fractal organization over multiple time scales from 30 s to at least 4.4 h, coexisting with synchronized ultradian rhythms. Using wavelet analysis we were able to show an overall temporal cascade formed by the progressive branching of maximum amplitude lines, in all animals studied (Fig. 4). This scale-invariant (fractal) dynamic pattern of quails’ locomotion throughout a week is highly reminiscent of the fractal geometry displayed by the branching of trees, pulmonary or blood vessels. Scale-invariant fractal structure in healthy cardiac dynamics was previously reported using wavelet analysis^33^.

Traditional analytical tools present difficulties for both detecting ultradian rhythms and discriminating them from harmonic frequencies of the circadian rhythm (i.e., frequencies integral multiples of the circadian rhythm)^24^. Indeed, Power Spectrum and Enright’s analyses were unable to detect all possible rhythms in time series, thus introducing differences between animals (Fig. 2). Locomotion time series are non-stationary (i.e., mean and variance change over time), which could hinder rhythm detection with analytical methods appropriate for stationary time series such as Power Spectrum^34^. Moreover, some animals showed unusually strong circadian rhythm (i.e. high daytime *vs*. very little nighttime locomotion) which could lead to underestimation of weaker ultradian rhythms (Fig. 2a). These methodological limitations can potentially introduce a large variability in the period of the ultradian rhythms that can be clearly detected for a given species, and even in a single experiment. Since a priori information about the presence of noise and stationarity in the time series is not always possible, a combination of different methods for detecting rhythms appears to be the most reliable approach.

In our study, wavelet analysis enabled the unambiguous and highly sensitive detection of ultradian rhythms of long (12 h, 8 h, 6 h, 4.8 h and 4 h) and short periods (2 h and 1 h) on an individual level, as already suggested from simple visual inspection of actograms (Fig. 1b). As a complementary method to wavelet analysis, EMD was applied to detect rhythms and visualize their waveform in the locomotor time series. The combined approach utilizing EMD and wavelet analyses, benefits from the strengths contributed by each analytical tool. By algorithmically interpolating points in the time series, using recursively a cubic spline function to generate data-dependent smooth oscillatory curves, EMD does not need to impose a mother wavelet. This feature is important when estimating characteristics of non-stationary time series, where the underlying waveform is unknown (i.e., they could be square, triangular, sinusoidal, etc). Without detrending or denoising of the time series, EMD successfully selected temporal scales corresponding to rhythm’s periods of roughly 24 h, 12 h, 8 h, 6 h and 4 h and lower (Fig. 3), and showed the smooth oscillatory nature of both circadian and ultradian rhythms. On the other hand, the complex morlet as the mother wavelet used to analyze the locomotor time series, takes advantage of the smooth oscillatory nature of the signal and is very efficient in detecting each rhythm (“unmixing modes”), in particular, for sinusoidal-like waveforms. In this case, the advantage of wavelet over EMD is given by the ability of the former to select the range of scales of interest for precise comparison between animals (Fig. 6). Additionally, in EMD applied to temporal scales corresponding to short time periods, the IMFs progressively include more than one frequency (Fig. 3) due to mode mixing^35^.

Although we present strong evidence in favor of the existence of ultradian rhythms and their smooth oscillatory nature, we are aware that all frequency-based methods utilized for rhythms detection (e.g. wavelets) potentially suffer from lack of specification, i.e. peaks from the frequency analysis may be the result of nonlinearity rather than ultradian rhythms^30^. To specifically address this issue we re-analyzed the time series with different wavelet families, such as the Frequency B-Spline (Supplementary Fig. 4a,b), Gaussian (Supplementary Fig. 4c) and Coiflets wavelet transforms (data not shown), in addition to the complex morlet wavelet used throughout this work, and obtained equivalent branching patterns.

Ultradian rhythmic locomotor activity with similar periods to those described herein has been previously documented in other vertebrate species^12, 13, 36–40^. For example, in wild-type mice significant peaks in amplitude of the modulus of the wavelet coefficient have been reported; these ultradian rhythms were distributed in the range of approximately 6.1 to 14.8 h but the number of peaks and their dominant frequency were not consistent across individuals^41^.

As far as we are aware, our report is the first to show consistent and reproducible rhythms in all animals over a broad time spectrum. Furthermore, the coherent and synchronized ultradian rhythms (Supplementary Fig. 6) described exhibit periods of a fraction of the circadian cycle, a chronobiological pattern that may represent an evolutionarily advantageous mechanism for keeping a constant phase relationship with circadian rhythms and major environmental periodicities^42^. Additionally, ultradian behavioral rhythms are often related to feeding behavior^43^. In this regard, autocorrelation analysis revealed that individually housed quails display ultradian feeding rhythms of approximately 45 min^44^ (24 h/32 = 0.75 h), which is consistent with the observed locomotor behavior. Locomotion synchronization within poultry social groups have already been reported^15, 16^, and in mammals it has been shown that only direct body contact induces social synchrony^14^.

An important finding of the present work is that not only ultradian rhythms with the same oscillatory periods were found in all birds (Fig. 6f), but also that a high level of synchronization of these rhythms among birds was found as well (Supplementary Fig. 6), irrespective of being tested while visually isolated from each other. Synchronization, defined herein as the correlation exhibited by the amplitude of the *Re*(*cwt*) at the corresponding time scale of each ultradian rhythm, was put in evidence as a positive correlation indicating peak amplitude coincidence between different birds. The fact that the two independent experimental groups studied displayed the same synchronization pattern (Supplementary Fig. 6) is in agreement with the existence of an endogenous, autonomous dynamic pattern. The rhythms synchronization between individuals is likely influenced by the light/dark cycle, which was the same for all animals and is known to be a powerful *zeitgeber* able to modulate neuroendocrine responses. The correlation of circadian rhythm between birds was maximal (≈1; Supplementary Fig. 6), while the correlation between ultradian rhythms was slightly lower. Since quails were visually but not acoustically isolated, some influence in this regard cannot be ruled out. Scheduled events^1^ during husbandry could also have an influence on behavioral synchronization but we were able to rule them out as zeitgebers by comparing results to a control experiment performed on completely undisturbed animals (Supplementary Figs 7–9).

Coordination in the ultradian domain is essential for biological time keeping^45^. Hildebrant *et al*.^4^ already proposed the endogenous, autonomous, nature of ultradian rhythms that keep internal order by means of frequency-and phase-coordination. Examples of faster rhythms or oscillations, cycling many times in a day in the range of an hour to milliseconds, abound in biological systems^7^. However, if circadian and ultradian rhythms are both important for biological timekeeping, then the key question becomes: how is synchrony achieved between disparate oscillators across different time domains and body locations? Based on the overall fractal architecture and inherent scale-invariance of the quail’s locomotion dynamics, we propose that they are inter-dependent with oscillatory ultradian rhythms. The fractal pattern consists of branching time periods of the different rhythms spanning from longer to progressively shorter periods at higher temporal resolution. Macroscopically, the circadian rhythm superimposes on the intrinsic fractal dynamics given that depends on the day-light cycle^46^, which in turn influences the sleep-wake cycle. This is consistent with previous data showing that the scale-invariant/fractal temporal structure in motor activity fluctuations does not significantly change with circadian phase or during a forced de-synchronization protocol^1^.

The highly suggestive fractal pattern displayed by the locomotor dynamic behavior of the quail led us to enquire about putative underlying neurophysiological correlates. This question appeals to the notion of endogenous regulatory mechanisms of scale-invariant dynamics across multiple temporal scales^3, 24, 29^. Evaluation of time series with DFA and frequency histograms of immobility duration events suggests that underlying fractal patterns in locomotor activity exist, which is consistent with previous shorter studies^18^. The stability of the observed power-law distribution over a large range of scales indicates that the underlying dynamic mechanism controlling immobility (vs. locomotion) have self-similar, scale-invariant, statistical properties across time scales^29^. Interestingly, the scale-invariant pattern of activity shown in quail (α ≈ 0.85) is similar to humans (α ≈ 0.90) and rats (α ≈ 0.89), supporting Hu *et al*.^24^ contention that a common intrinsic control mechanism across species could exist. Fractal, power-law distribution of immobility events over a broad range of scales as those presented herein have also been reported in other taxa, e.g., Drosophila^47^, mice^20, 48, 49^, and humans^20^.

Bird species have at least three autonomous and anatomically distinct oscillators in retina, pineal gland, and hypothalamus that have been proposed to organize circadian pace making^50^. Mammals with lesions in the circadian pacemaker (suprachiasmatic nucleus, SCN) can keep behavioral ultradian rhythmicity such as feeding in vole^41^ and sleep in rats^51^. However, more recent studies show that circadian and ultradian rhythms in rat locomotion are almost completely abolished after SCN- lesion in agreement with its critical involvement in the scale-invariance (16 min to 24 h), and strongest influence at longer time periods (>4 h)^24^. Further support is provided by data from humans where aging and Alzheimer’s disease (both associated with progressive dysfunction of the SCN) significantly attenuate the scale invariance of arm activity fluctuations^52^. Fluctuations in stride interval during walking also present long-range (fractal) correlations that degrade during physiological aging and certain neurodegenerative diseases such as Huntington and Parkinson^23, 53^ although they are independent from scheduled and environmental influences^29, 54^.

The circadian system may play a central role in fractal regulatory networks responsible for the temporal organization of motor activity^3^ considering that, at least in rats, the SCN modulates both circadian and ultradian rhythms in addition to scale-invariant patterns^24^. Consistent with present findings, these observations indicate that ultradian rhythms interrelate in a fractal branching pattern. We conjecture that this pattern emerges from a generalized mechanism consisting of a control network^3^ of biological oscillators spread over multiple locations and functional temporal scales, interrelated nonlinearly through coupled feed-back loops. The SCN would be a major node from the neural network in mammals, as the retina, pineal gland and hypothalamic oscillators would be in avian species^3, 24^. Clearly, more work is needed to investigate this possibility.

In summary, we report for the first time that the overall underlying dynamic organization of locomotor time series from quails is fractal, presenting scale-invariant dynamics spanning at least three orders of magnitude (from seconds to hours) that coexist with ultradian rhythms. All birds exhibit synchronized ultradian rhythms and fractal self-similarity. Wavelet analysis unveiled the fractal architecture of the overall locomotor dynamics as a self-similar branching pattern. These findings are in agreement with the idea that the distinctive pattern found is an emergent property from a network of oscillators, functioning in multiple temporal scales at different spatial locations, nonlinearly interrelated through feed-back loops. This chronobiological pattern could represent an evolutionarily advantageous strategy for keeping the organism’s endogenous rhythms in phase with internal and environmental periodicities, notably the feeding, light-dark and sleep-wake cycles.

## Materials and Methods

*General Procedure:* animal husbandry followed standard procedures described in section SI Animals and Husbandry (see Supplementary Material). All the procedures were in compliance with the *Guide for the Care and Use of Laboratory Animals* issued by the National Institute of Health (NIH Publications, Eighth Edition). The experimental protocol was approved by the Institutional Committee for the Care and Use of Laboratory Animals (Comité Institucional para el Cuidado y Uso de Animales de Laboratorio (CICUAL)) of the FCEFyN - National University of Cordoba.

A detailed description of the experimental setup is described elsewhere^55^. Briefly, animals were housed in a white wooden box measuring 40 × 40 × 40 cm (width × length × height, respectively) with a wire-mesh floor. A metal bar wall divided the box in two same-size zones (feeding, nesting), and an opening served as a door allowing the bird to move freely between compartments. In the feeding zone, a feeder and an automatic nipple drinker were positioned, while in the nesting zone, a small rubber nest measuring 15 × 15 cm was placed. Nylon monofilament line was extended over the top of the boxes with a 1 cm separation in order to prevent the birds from escaping without interfering with their visualization. A video camera was suspended 1.5 m above the box. These cameras have built-in, infrared LED lighting, automatically switching to infrared recording after lights were turned off. The camera was connected to a computer in an adjacent room that could be accessed remotely by experimenters.

The locomotor activity of 24 adult quail (100–140 days old) was evaluated. Testing began at approximately 1:00 PM, when 12 quails were placed in a box, and transported to a nearby experimental room. Each bird was then placed individually in the feeding zone of one of the boxes. During the following 6.5 days, the birds’ locomotor activity was continuously recorded. Testing ended at 9 PM the 7^th^ day. Daily housekeeping activities (egg collection, cleaning and feeding) were performed between 12 and 12:30 PM; the activity during this period was not included in time series, which in total implied a loss of <2% from the data set. It should be noted that Ma *et al*.^56^ studied the effect of data loss on the estimation of α with DFA, and showed that for 0.7 < α < 1, global scaling exponents remained unchanged for data loss up to 65%^56^.

Since only 12 birds could be tested simultaneously, two consecutive groups of 12 individuals were evaluated. We used the ANY-MAZE@ computer program to register locomotion at 0.5 s intervals (x_i_). The locomotor time series (1.07 × 10^6^ time intervals) of each bird was obtained by assigning a number one (x_i_ = 1) if during the interval the bird was ambulating, or a zero (x_i_ = 0) if immobile. Locomotor time series are publicly available at FigShare (http://dx.doi.org/10.6084/m9.figshare.1424729) and a complete description of time series and software used is described in Guzman *et al*.^55^.

For each bird, *percentage of time spent ambulating* was estimated as: Amb_%_ = (Σx_i_/N)*100, where N is the total number of intervals in 1 h (7200 time intervals/h). Similarly, *actograms* were computed for each bird by estimating the percent of time ambulating in 6 min bins (720 time intervals/bin).

*Fourier analysis* (also called Power Spectrum analysis): in this method a periodogram is constructed, and if there is oscillatory behavior in the data set, its period will show up as a peak in the spectral energy^24^. We used the Fast Fourier Transform (FFT) subroutine of MATLAB to perform power spectral analysis on the time series. The power spectrum is widely used for measuring correlations in stationary time series^55^. The fact that the log-log plot of the power spectrum *S*(ƒ) *vs*. ƒ is linear implies, *S*(ƒ) ~ ƒ^−β^. The exponent β is related to the mean fluctuation exponent α by β = 2α – 1, serving as an indicator of the presence and type of correlations^33^.

*Enright*’*s method:* also referred as the “χ^2^ periodogram”, is based on the principle that if we break down a periodic time series into blocks of period *P*, then the blocks will be very similar to each other if the data set actually has period *P*, but not if the set has a different period. Thus, differences between blocks can be used as an index of how well the chosen period approximates the real period^57^. To reduce the impact of noise in the analysis, we applied the Enright’s method to actograms. The *Q*_*P*_ statistic (i.e. an index of how periodic a data set is) were estimated for all possible blocks of *P* points, where 1 ≤ *P* ≤ N/2. We calculated the local maximum of the periodogram and sorted them out in ascending way all periods with *Q*_*P*_ values higher than 10% of the maximum *Q*_*P*_ values found in the periods. Since Enright method does not identify harmonics in time series, every multiple of the periods found in the previous step is removed^58^, thus limiting the possibility of detecting ultradian rhythms multiples of each other. The open source code described in ref. 58 was used for analysis.

*Empirical mode decomposition (EMD):* proposed by Huang *et al*.^59^ is a data-driven algorithm that decomposes signals into Intrinsic Mode Functions (IMFs) which admit an unambiguous definition of instantaneous frequency and amplitude at each sampled time for each component. In such decomposition, the signal may be seen as fast oscillations superimposed to slow oscillations, with iteration on the slow oscillations considered as a new signal^60^. This method considers that “fast” and “slow” components are not predefined through a filtering operation but according to an algorithm similar to an adaptive filter bank. Herein, EMD analysis was performed on all time series, without detrending or denoising the signal, utilizing the open source package emd (available at http://perso.ens-lyon.fr/patrick.flandrin/emd.html). To reduce the impact of noise in the analysis, while keeping a large number of data points for period estimation, EMD was performed with 30 s bins of ambulation time series (18830 data points).

*Wavelet analysis:* This analytical approach allows a signal to be decomposed in a flexible manner, providing simultaneously information on the presence of periodic behavior and its time localization^31, 61, 62^. Herein, we used the complex (*cwt*) Morlet wavelet (Supplementary Fig. 1) in the first windowed Fourier transform to extract time-dependent features. When using a complex waveform, its transform is also complex, thus, the *cwt* coefficients can be represented by their real and imaginary parts, or amplitude and phase angle. Data analysis in this article was done using the wavelet toolbox of MATLAB R2013a, in particular the continuous wavelet transform function, *cwt*. We used the complex Morlet wavelet, *cmor1-1.5*, with scales that ranged from 1 to 400 corresponding to periods of 0.06 to 26 h which has previously been used to study circadian and ultradian rhythms in mice^41^. For convenience, scales were transformed into frequencies using the *scales2freq* function of MATLAB. In order to reduce noise, wavelet analysis was performed on actograms with 6 min bins (Supplementary Fig. 2). Script is publically available^55, 63^. For the synchronicity analysis, we followed Pering *et al*.^31^ using the real part of the *cwt* (*Re*(*cwt*)). For additional information see section SI Wavelet analysis.

*Detrended fluctuation analysis (DFA):* the method utilized herein to determine scale-invariance and to evaluate the presence and extent of long-range correlations in the animal locomotor activity, was introduced by Peng *et al*.^64^ and is described in detail elsewhere^18, 55^ and in section SI Detrended Fluctuation Analysis. Briefly, DFA estimates the self-similarity parameter α that measures the autocorrelation structure of the time series. If α = 0.5, the series is uncorrelated (random) or has short-range correlations (i.e., the correlations decay exponentially), whereas 0.5 < α < 1 indicates long-range autocorrelation (i.e., correlation decays as a power-law), meaning that present depends on past behavior^32^. Also, α is inversely related to a typical fractal dimension, so in this case, the value increases with increasing regularity (or decreasing complexity) in the time series. This software is also available in the public domain (http://www.physionet.org/physiotools/dfa/) and the Matlab script used herein is publically available^55, 65^. Herein, DFA calculations were performed with a customized script ran on MATLAB R2013a.

Trends within the locomotor time series were also systematically studied^32^. A DFA of third order was the lowest detrending order that eliminated trends in all series and therefore it was applied to all series for estimating α (Supplementary Fig. 10). In addition, the appropriate scaling range was determined using the following criteria: stable values of local slopes, maximum coefficient of variation and minimum sum of squared residuals^18, 66^. This analysis showed that results above 4.4 h are not statistically reliable in 17% of the animals (see Supplementary Fig. 11 for details).

As a control of the detrending capacity of DFA to eliminate the slow oscillatory behavior of circadian rhythms, time series were filtered using a mean moving average with a period of 23.5 h and then reanalyzed with DFA for comparison to the analysis of unfiltered (original) data time series (Supplementary Fig. 12).

*Frequency distribution of the duration of immobility events* (*FDD-I*)*:* an immobility event was defined as an interval of time (>1 s) in which the animal remains immobile. To determine the duration of immobility events we used the complete time series, where the high resolution 0.5 s sampling rate corresponds to the resolution of a step. For each animal the FDD-I was analyzed by plotting the frequency *vs*. the duration of immobility events, using double logarithmic scale. Achievement of a linear fit was considered as an indication of a power-law (fractal) distribution, and its slope is known as the scaling factor, S^18^. In order to avoid the tail of the power law distribution in the calculation of the linear slope of the frequency histogram, only durations of events ≤ 250 s were included in the estimation of S (Supplementary Fig. 14). In contrast to immobility events, the frequency distribution of mobility events (FDD-M) did not show a clear power-law distribution (Supplementary Fig. 13). Since bin size can potentially affect not only the S value of the distribution but also the type of distribution, in Supplementary Figs 14 and 16 we evaluated the effect of bin size on FDD-I and FDD-M, respectively. Briefly, in the case of FDD-I, for bin sizes between 0.5 and 60 s, a power-law distribution was observed, although the exact value of S is dependent on bin size, showing a sharp increase for sizes larger than the mean immobility event duration (24 ± 2 s). In contrast, in the FDD-M case, an increase in bin size leads to a change in distribution, trending to a power-law (see Supplementary Information for further discussion).

## Electronic supplementary material

Supplementary Information
