# Supplementary material for: The fractal organization of ultradian rhythms in avian behavior
Source: Sci Rep. 2017 Apr 6;7:684. doi: 10.1038/s41598-017-00743-2 (PMC5429634; doi:10.1038/s41598-017-00743-2)
Supplement: Supplementary file 1 — Supplementary Information [file 41598_2017_743_MOESM1_ESM.doc]

**The fractal organization of ultradian rhythms in avian behavior**

Diego A. Guzmán^1,2^, Ana G. Flesia^3^, Miguel A.Aon^4^, Stefania Pellegrini^1^, Raúl H. Marin^1^, Jackelyn M. Kembro^1*^

^1^ Instituto de Investigaciones Biológicas y Tecnológicas (IIByT-CONICET), and Instituto de Ciencia y Tecnología de los Alimentos, Cátedra de Química Biológica, Facultad de Ciencias Exactas, Físicas y Naturales, Universidad Nacional de Córdoba , 1611 Vélez Sarsfield, X5016GCA, Córdoba, Córdoba, Argentina. ^2^ Deptartment of Animal Science, Aarhus University, 20 Blichers Allé, Post Box 50, DK-8830, Tjele, Denmark.^3^ Centro de Investigaciones y Estudios deMatemática (CIEM - CONICET), and Facultad de Matemática, Astronomía y Física FAMAF, Universidad Nacional de Córdoba, Ing. Medina Allende s/n Ciudad Universitaria, Córdoba, CP X5000HUA. ^4^ Johns Hopkins University School of Medicine, 720 Rutland Avenue, Ross Bldg. 1059, Baltimore, MD, 21205, USA.

**Supplementary Information**

**SI Animals and Housing**

Eighty mixed-sex Japanese quail hatchlings were randomly housed in 2 white wooden boxes (40 quail each) measuring 90 x 80 x 60 cm (length x width x height).At 28 days of age, female quail were randomly cage-housed in pares in a 6-tier cage battery units, where each cage measured 40 x 20 x 21 cm (length x width x height) following standard procedures described elsewhere ^1^. Quail were subjected to a daily cycle of 14 h light (300 to 320 lx):10 h dark during the study, with lights on from 6am to 8pm. These experimental conditions were kept constant throughout the experiment. Due to management practice, females were exposed to a brief (10 min) male-female sexual interactions up to 10 days prior to testing.

**SI Actograms**

The median 24-h actogram for each animal is displayed in Supplementary Fig. 1 which clearly shows peaks in addition to the circadian 24 h day/night cycle in all animals studied. The result obtained is in stark contrast with the expected one from a signal composed exclusively of circadian oscillations concomitant with random fluctuations, given that random fluctuations would have been smoothed by the median values over the 6 day period. Although this figure is inappropriate for evaluating periodicities, it provides clear evidence of peaks colocalization in the actograms of each one of the animals analyzed.

A

B

C

D

**Supplementary Figure 1:** Estimated median actograms from 6 min windows over 6 days of testing. Colored lines in the panels denote each one of the 24 animals studied represented in the different panels as follows: A) 5,B) 6, C) 5, and D) 8 animals. Median values were plotted to avoid the impact of extreme daily values on estimation. To facilitate visualization, the time series of one of the animals is represented with a thicker black line.

**SI Wavelet analysis**

Wavelet analysis expresses a signal as a sum of component waveforms. Whereas the waveforms in Fourier analysis are sines and cosines of a set of fixed frequencies, wavelets are not periodic but localized in different frequency bands. This allows signal decomposition in a more flexible manner improving time localization^3, 4, 5^. Another advantage of wavelet analysis is the flexibility for choosing the mother wavelet according to the data set. Orthogonal wavelets are used to detect sharp singularities, while local periodic behavior is better detected with complex wavelets or analytic ones (wavelets with real Fourier transform). The Morlet family has a complex mother wavelet (also called Gabor wavelet) composed of a [complex exponential](http://en.wikipedia.org/wiki/Complex_exponential) [carrier](http://en.wikipedia.org/wiki/Carrier_wave) multiplied by a [Gaussian window](http://en.wikipedia.org/wiki/Gaussian_window) (envelope). This wavelet is widely used for detection of local periodic behavior.

When using a complex waveform like the Morlet wavelet, its transform is also complex. Thus, the *cwt* coefficients can be represented by their real and imaginary parts, or amplitude and phase. When selecting the Morlet wavelet as a mother wavelet, the phase shift between real and imaginary parts allows us to eliminate the wavelet’s oscillations when visualizing the coefficient modulus (Supplementary Fig. 2). From the resulting complex valued coefficients we can separate the modulus that give the energy density and the phase which detects singularities and measure instantaneous frequencies^6^.During the analytical process, this wavelet is rescaled for each time scale (period) evaluated (“y” axis in Fig. 3-5 and Supplementary Fig. 3).

**
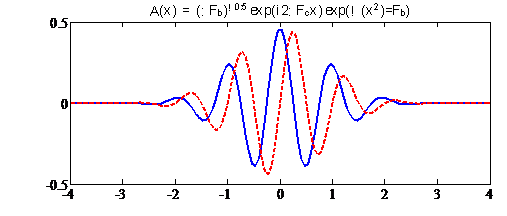
**

**Supplementary Figure 2: Example of the shape of the Morlet wavelet.** Real (solid blue line) and imaginary (dashed red line) part of the Morlet wavelet with parameters bandwidth, Fb = 1.5, and center frequency, Fc = 1.

Wavelet analysis was first performed on the complete time series (Supplementary Fig. 3). Although the periodic nature of the signal can be visualized the output is noisy. This noise was reduced significantly when wavelet analysis was performed on actograms with 6-min bins (Fig. 4), and for this reason wavelet analysis of actograms was performed for detection of circadian and ultradian rhythms in animal activity patterns.

**
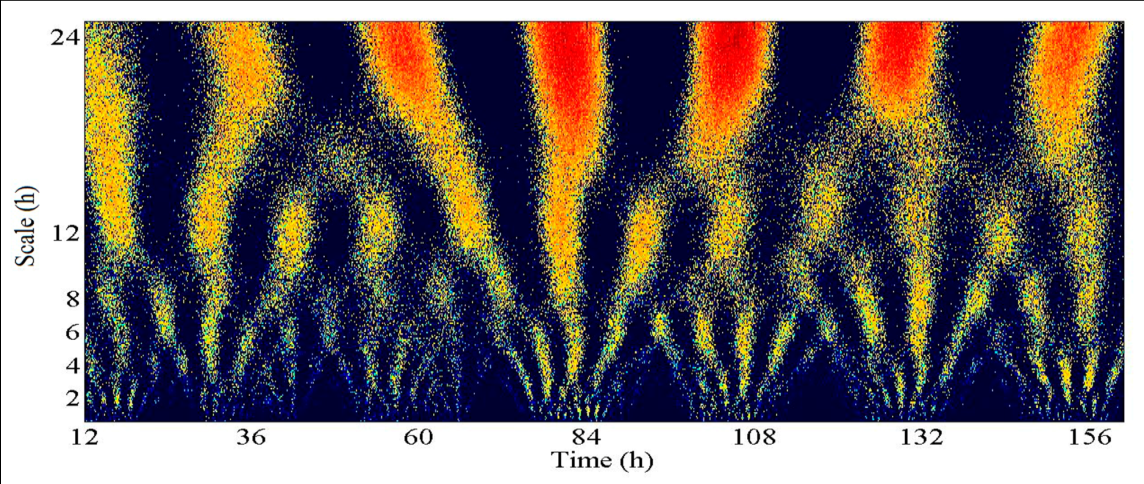
**

**Supplementary Figure 3: Example of amplitude plots of the real coefficient of the complex valued Morlet transform.**

Wavelet analysis was performed on the complete data set. When compared to Fig. 4 in the main text, where wavelet analysis was performed on actograms with 6-min bins, the same ramification pattern is observed but with a noisier output.

To determine that the branching pattern described by the wavelet coefficients is not an artifact of the detection method (based on a plot of the coefficients of the continuous complex Morlet wavelet transform), the time series was reanalyzed using different wavelet families (Supplementary Fig. 4A) including the complex Frequency B-Spline (Supplementary Fig. 4B,C), and two real families, First Derivative Gaussian (Supplementary Fig. 4D) and Coiflets wavelet family (data not shown), in addition to the Complex Morlet wavelet presented in the main text.

Notice that the branching pattern in the phase plot is observed independently of the wavelet family used (Supplementary Fig. 4C,D) including the shape and definition, irrespective of the fact that all wavelet families utilized are quite different. As known, the wavelet transform essentially measures the similarity between the signal to be analyzed and the shifted and scaled version of the base wavelet. In this sense, an appropriate wavelet can be chosen selecting the set of hypotheses over the smoothness space where the analyzed signal belongs. For example, a train of impulses added to fractal noise must be studied with the Haar family, or a superposition of smooth waves localized in time with the bumps wavelet family.

The complex Morlet and B-spline families are more appropriate for the study of a sum of periodic sinusoidal waves. Nevertheless, the presence of ultradian rhythms must be detected with all transforms despite the fact that not all of them will exhibit the same degree of success in localizing the correct period. As a matter of fact, several cross-sections of the relative intensity of the First Derivative Gaussian wavelet transform coefficients are plotted (Supplementary Fig. 4E) showing different time scales for comparison. A similar image was observed using the Complex Morlet Wavelet Transform (Supplementary Fig. 5).

Periodic peaks in coefficients of the First Derivative Gaussian wavelet appear at the 24, 12, 8 and 6h time scales. Within this framework, we chose the Complex Morlet wavelet for the analysis of our data in the original manuscript because it is widely used for detection of sinusoids embedded in noise. Moreover, wavelet coherence and correlation concepts used in our manuscript were developed with the Complex Morlet 1,2.

An example of quantification of circadian and ultradian rhythms of period 24, 12, 8, 6, 4 and 3 h is represented in Supplementary Figure 5. The number of peaks in the relative intensity of the real wavelet transform coefficient observed at the respective scales (7, 13, 19, 25, 30 and 49 peaks, respectively), and the estimated lag times between successive peaks (23.3, 11.9, 7.9, 5.7, 3.9 and 2.9 h) provide analytical confirmation of the presence of these rhythms. For smaller scales of periods <2 h, ultradian rhythms could also be quantified by peak counting and lag estimation.


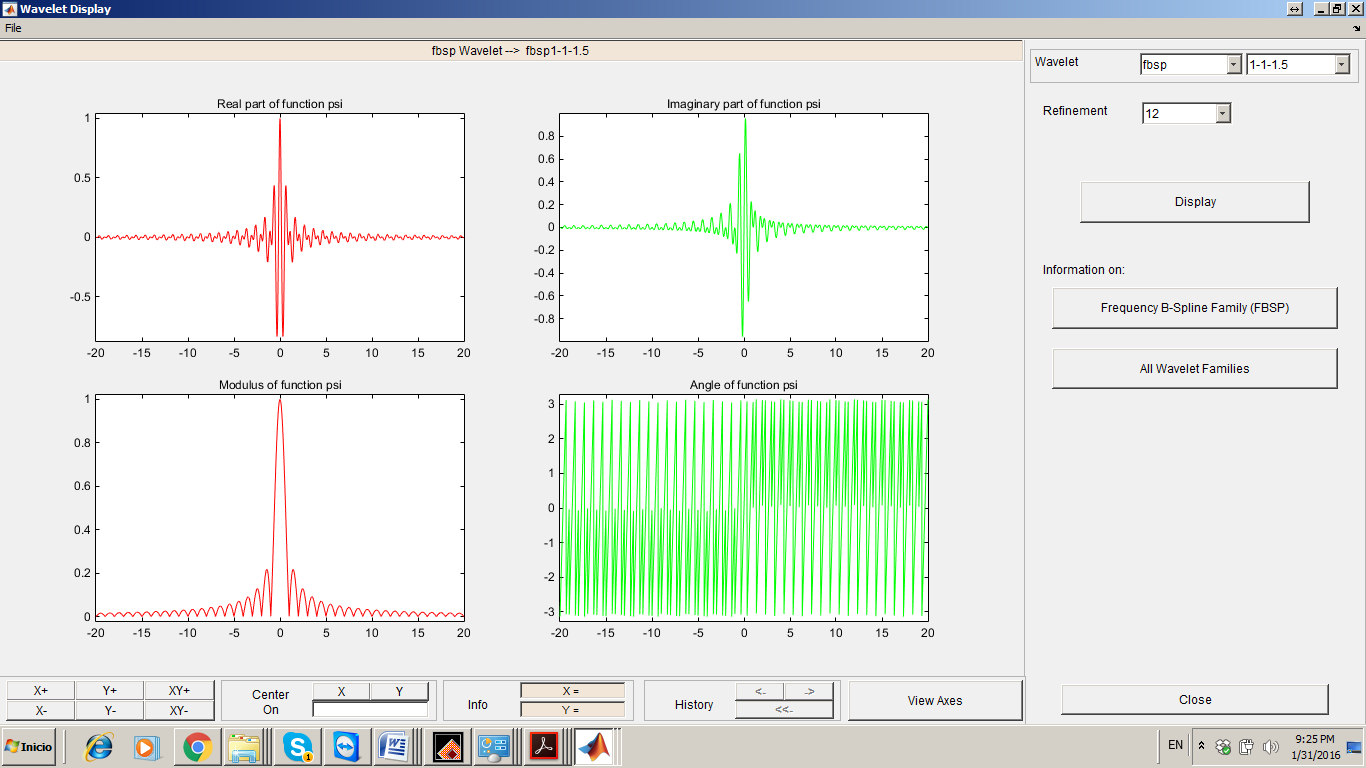

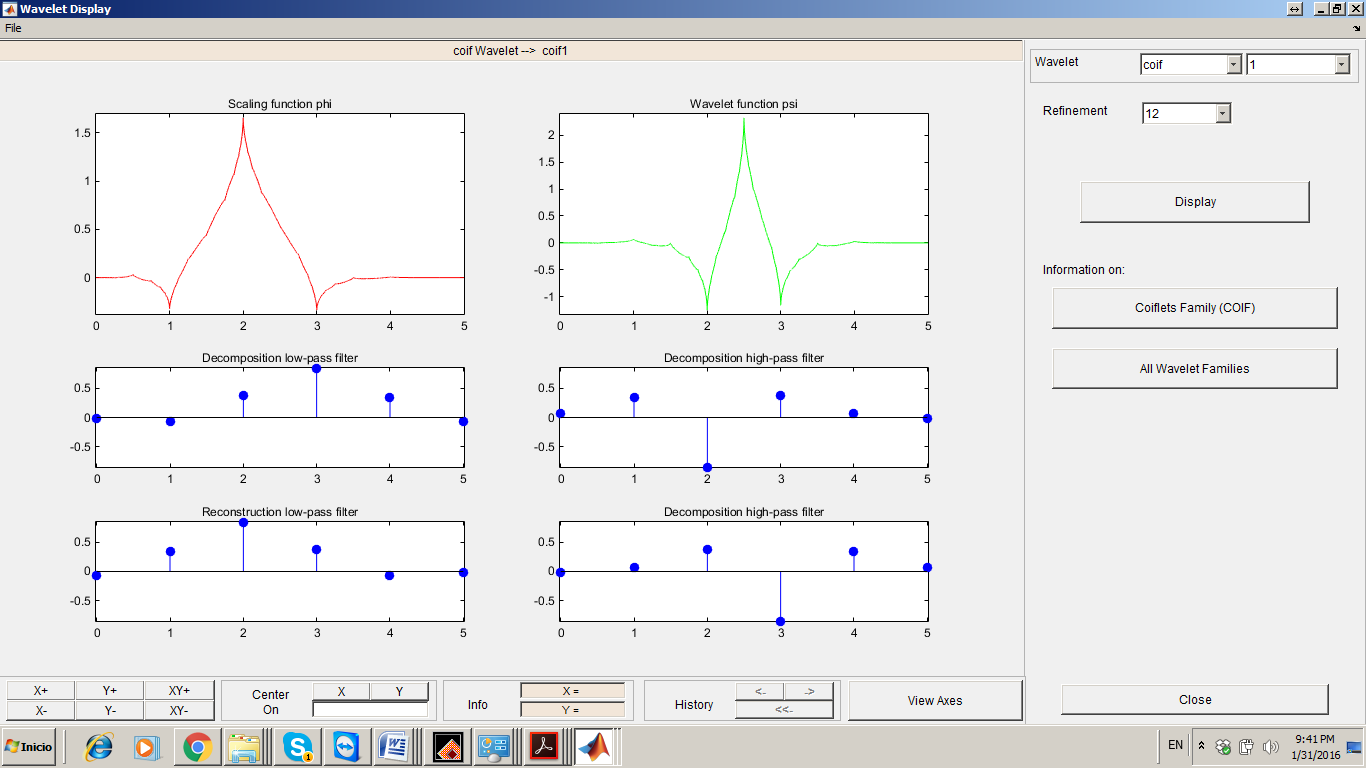

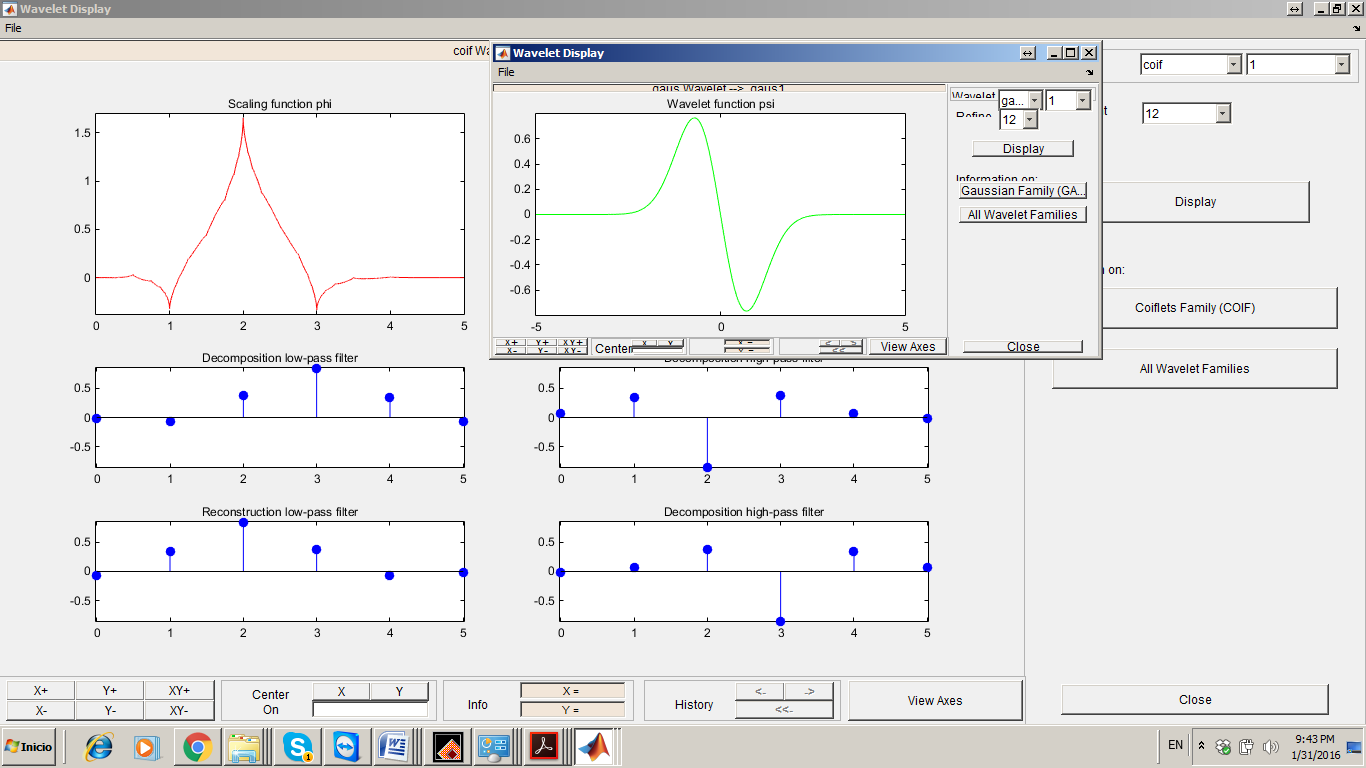

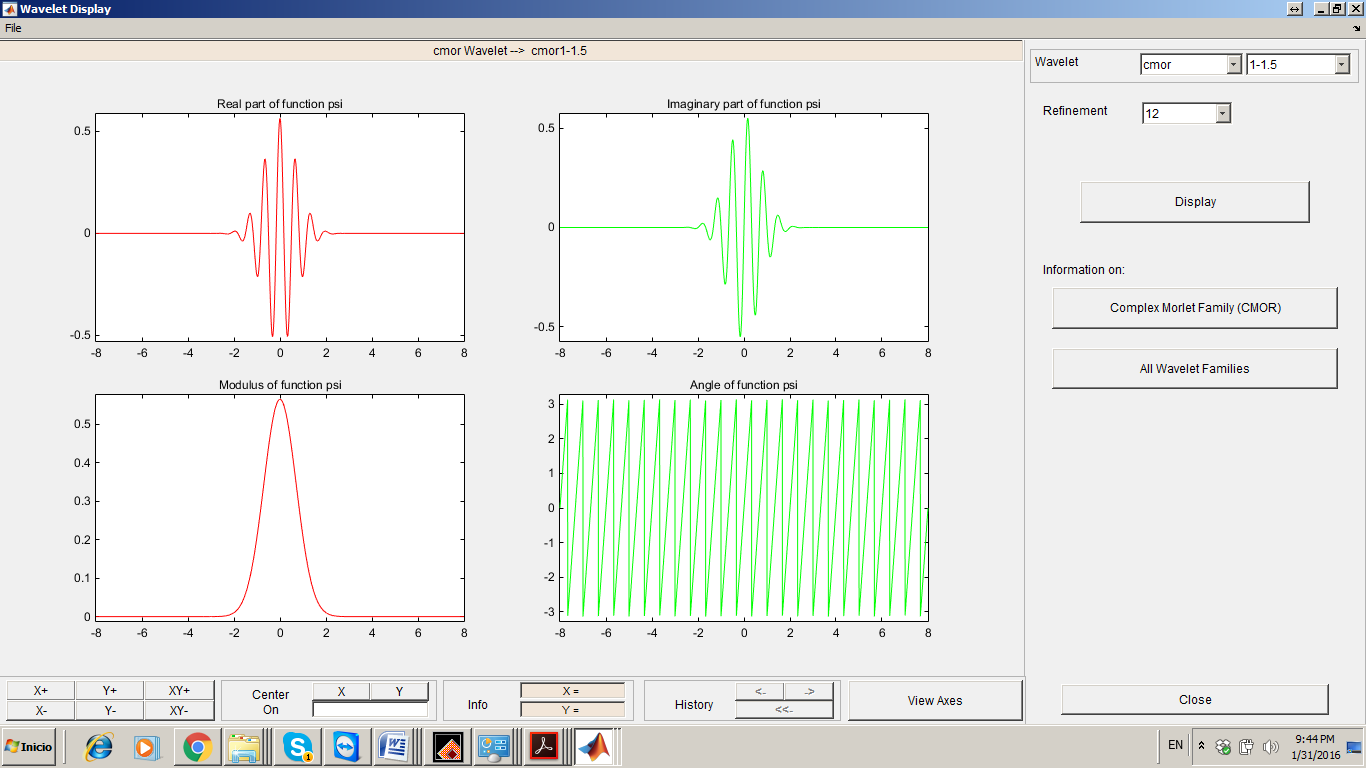


A

*
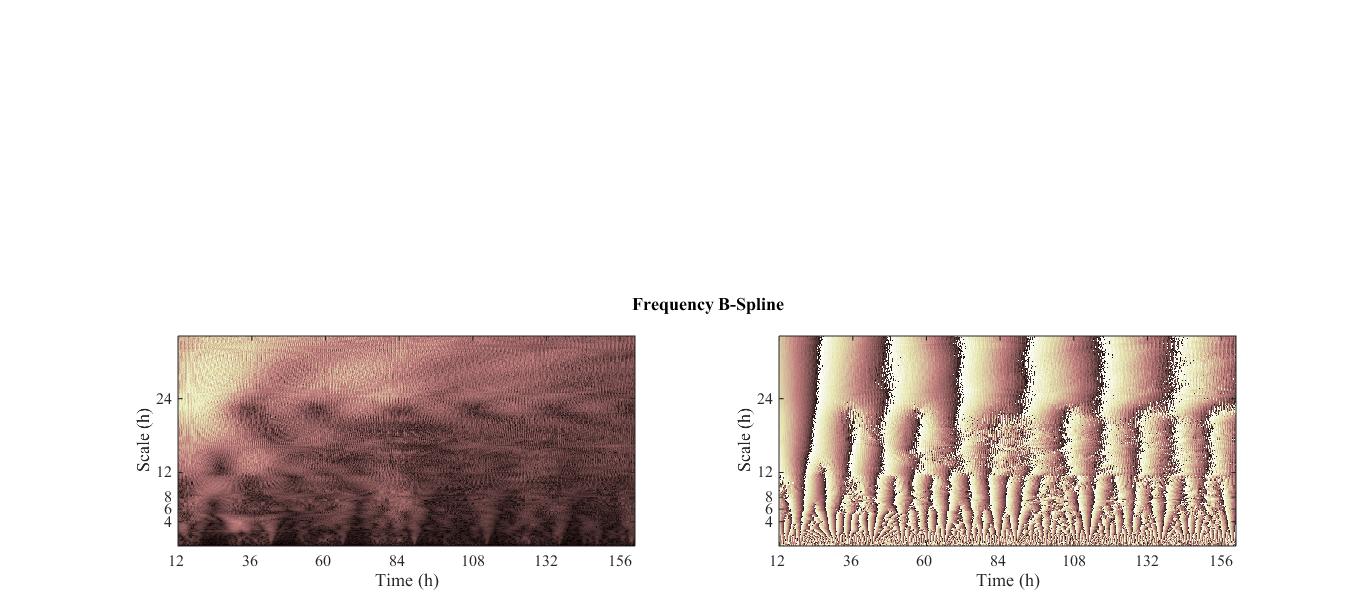

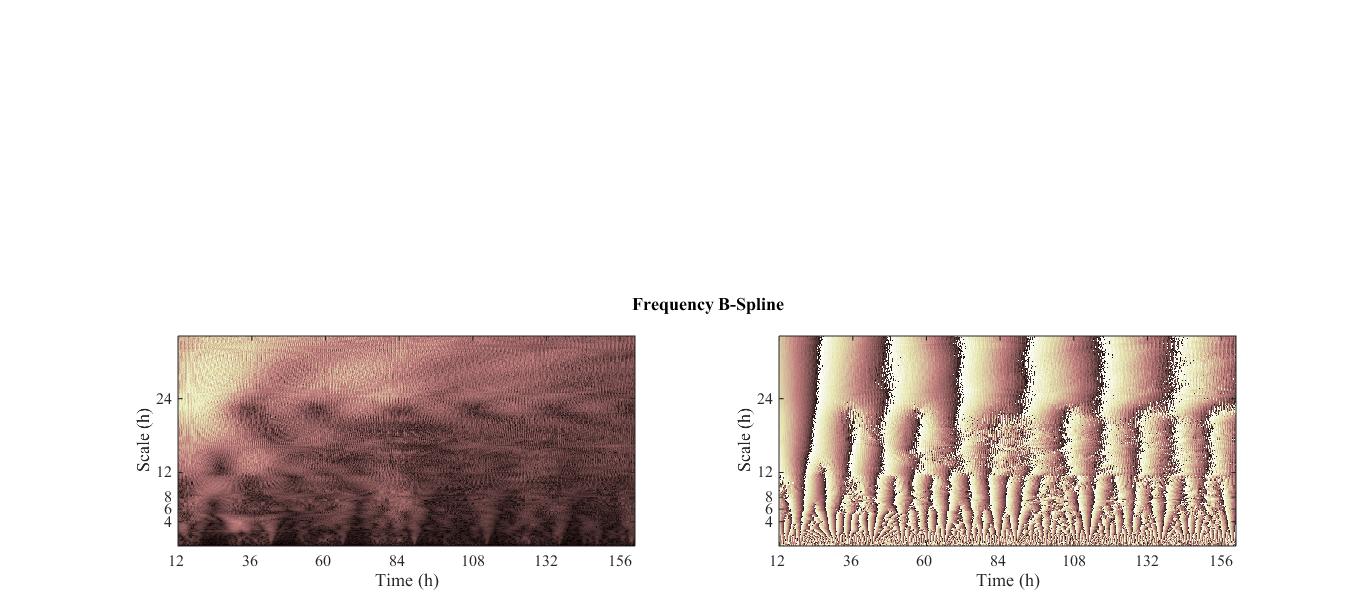
*

B

C

E

D

**Supplementary Figure 4: Wavelet analysis of locomotor actograms using Frequency B-Spline and First Derivative Gaussian wavelets.** A) Representation of the real part of the four wavelet transforms used to analyze the data, i.e. the Frequency B-Spline (FBSP), First derivative Gaussian (Gaus1), Coiflets, (Coif) and the Complex Morlet (C. mor).

FBSP and C. mor are complex wavelets (only real part is represented in panel in green), while Gaus1 and Coif are real (represented in red). Amplitude plots of B) modulus and C) phase angle using a Frequency B-Spline (Matlab wavelet name=fbsp1-1-1.5, scales used 1:1:350). D) Amplitude plot of the real coefficients using a Gaussian wavelet (Matlab wavelet name=gaus1, scales used 0 1:0.1:66). Wavelet analysis was performed on actograms with 6-min bins, and the same ramification pattern is observed for both wavelet transforms (compared to Fig. 4 of the manuscript which was estimated using the C. mor Wavelet. E) A cross-section of the relative intensity of the real wavelet transform coefficient shown in "D" at different time scales is represented.

**
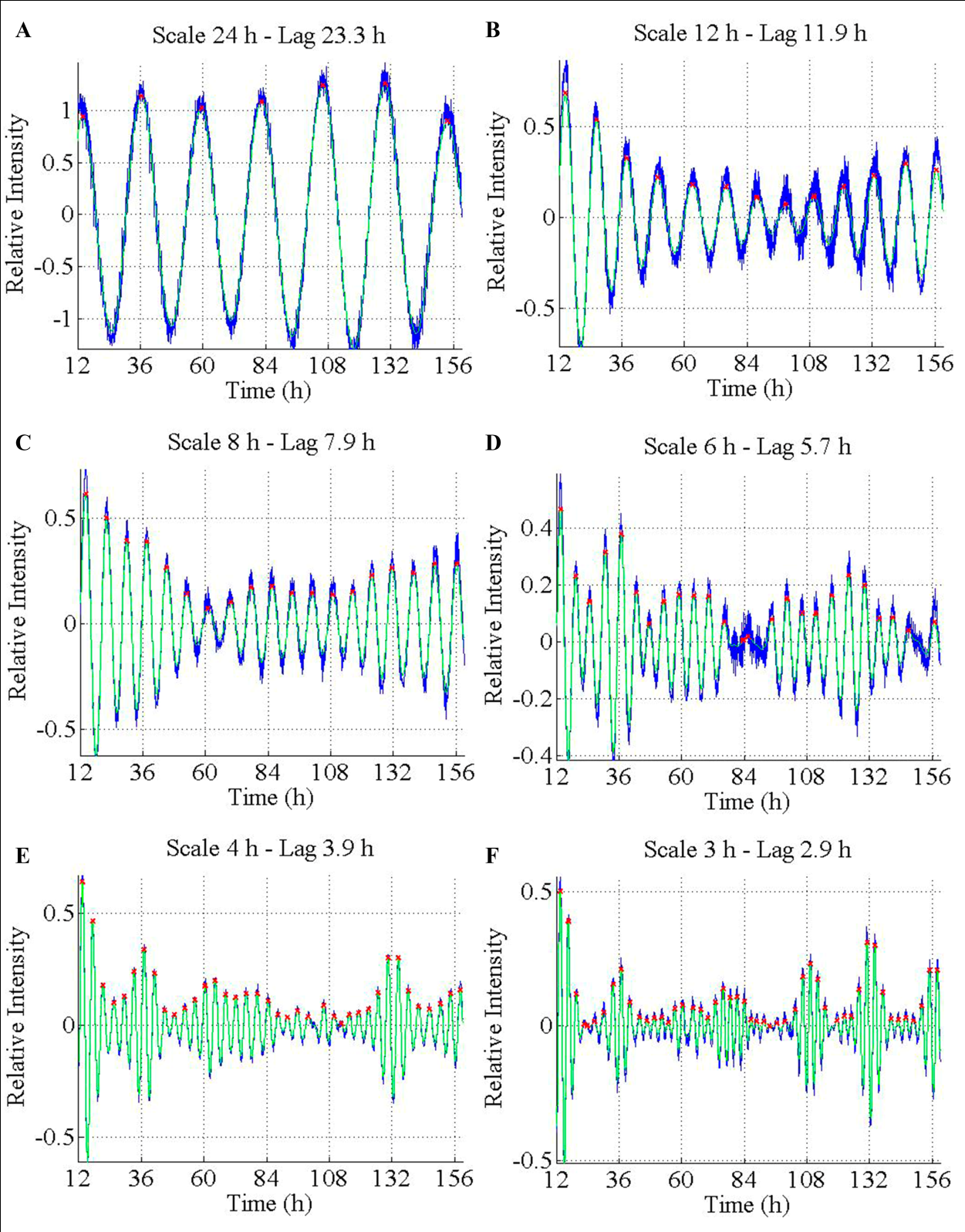
**

**Supplementary Figure 5: Estimation of intensity peaks of real wavelet transform coefficient and time lag between peaks.**

A cross-section of the relative intensity of the real wavelet transform coefficient shown in Fig. 5D at different time scales is represented. Peaks in the relative intensity provide information on the presence of the oscillation of the corresponding period throughout the 6.5 days of testing. A) At the 24 h period scale, 7 peaks corresponding to the daily circadian rhythm are observed, while at the 12 h period scale 13 peaks can be detected (2 peaks per day). For smaller scales of period C) 8h, D) 6h, E) 4h and F) 3h, the number of peaks observed are 19, 25, 30 and 49, respectively. The estimated mean time lag (“Lag”) between peaks was calculated.

Supplementary Figure 6 shows the mean value of the correlation coefficient obtained at each time scale from all 60 pairwise comparisons between 12 animals from the same experimental group. As expected at 24h scale, a high mean correlation coefficient was observed, given that all animals were under the same photoperiod. Interestingly, peaks in the mean correlation coefficient were also observed at12, 8, 6 and 4 h, indicating that these ultradian rhythms could be synchronized in all animals with respect to the time of day they appear.


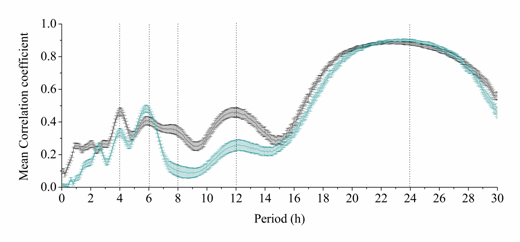


**Supplementary Figure 6: Synchronization between animals in ultradian rhythms in locomotor activity.**

Pairwise comparison of the real wavelet coefficients between animals at each time scale was performed using the Spearman correlation coefficient. Mean ± Standard Error is represented of all pairwise comparison between all 12 animals from the same experimental group. The two experimental groups (black and blue lines) showed similar patterns. Wavelet analysis was performed on actograms with 6-min bins in order to reduce noise.

**SI Control experiment with undisturbed quails**

Since daily maintenance activities could potentially result in the appearance of ultradian rhythms, we performed an independent experiment in which the individual quails remained completely undisturbed while their locomotion activity was being monitored during 5.5 days. In this experiment wood shaving instead of a wire mesh floor was used as bedding to avoid dailycage maintenance. Feed and water were provided ad libitum. Animals and Husbandry followed standard laboratory procedures, and the same conditionsas in the main experimental setup were used (except for bedding). Birds were allowed a 7 days habituation period to the new environment before being tested.

Supplementary Fig. 7 shows the PSA performed on all 4 animals with the corresponding peaks in 24, 12, 8, and 6 marked with grey doted lines, higher frequency peaks can also be observed. An example of EMD is observed in Supplementary Figure 8, and sinusoidal IMFs (IMFs 11-13) were found that clearly distinguish circadian and ultradian rhythms of approximately 12h, 8h and 6h, while smaller IMFs showed mode-mixing, similar to results now presented in Fig. 3 main text.

The wavelet analysis of the time series of locomotor activity in the control experiment were similar to those found in the experiment presented in the main text (Figs. 1, 4), ruling out daily maintenance activity as a zeitgeber. In Supplementary Fig 9 an example of quantification of circadian and ultradian rhythms of period 24h, 12h, 8h, and 6 h is shown. The number of peaks in the relative intensity of the real wavelet transform coefficient observed at the respective scales (5, 11, 17, and 22 peaks, respectively), and the estimated lag times between successive peaks (24h, 12.01h, 8.08h, and 5.8h) provide analytical confirmation of the presence of these rhythms. For smaller scales of periods < 6h, ultradian rhythms could also be quantified by peak counting and lag estimation. A visual representation of the sum of the relative intensity of the real wavelet transform corresponding to the time scales 24h,12h, 8h and 6h was superimposed on the actogram is shown in the bottom panel. Note how, although lacking addition scales, this method is capable of revealing maximum peaks in actograms. Together, the results of this control experiment show that the pattern of rhythms exhibited by undisturbed birds present the same periods as those obtained in the main experiment, providing further evidence of the presence of ultradian rhythms in locomotor activity, and of their endogenous origin rather than the result of entrainment by exogenous factors other than light-dark, sleep-wake and feeding cycles.

**Supplementary Figure 7: Synchronization between animals in ultradian rhythms in locomotor activity.** Power spectrum analysis of the locomotortime series of 4 control quails (each represented by a different color) which remained undisturbed during the totality of the experiment. Dotted lines mark frequencies corresponding to the 24h, 12h, 8h, and 6h periods.

A

B

**Supplementary Figure 8:**.**Empirical Mode Decomposition(EMD) of ambulatory activity time series of control undisturbed quail.** A) EMD of the actogram constructed using 30 sec window (top panel in blue). For visualization of the 17 IMFs found, only 10 are shown (Imf 5 - 13). B). The Power Spectrum Analysis (PSA) of IMFs 9-13 are depicted in the right panel.

The 30 sec window for EMD was selected as a tradeoff between the need for a large number of data points for PSA while taking advantage of the possibility of smoothing the time series by pooling data in larger time windows.

A B

C

**Supplementary Figure 9: Wavelet analysis of ambulatory activity time series corresponding to undisturbed birds during 5.5 days.** Amplitude plots of complex valued Morlet transform: a) Modulus, and b) phase angle. The *x*-axis represents time (5.5 days) and the *y*-axis denotes the scale of the wavelet used (from 6 min to 26 h). The wavelet decomposition reveals an overall self-similar fractal structure particularly noticeable at low scales, while the periodic behavior of the series is more clearly depicted at large scales. Similar plots were obtained for all 4 animals studied. c) Top panels (magenta) represent cross-section of the relative intensity of the real wavelet transform coefficient at different time scales. Peaks in the relative intensity provide information on the presence of the oscillation of the corresponding period throughout the 5.5 days of testing. At the 24 h period scale, 5 peaks corresponding to the daily circadian rhythm are observed (estimated lag between peaks of 24h), while at the 12 h period scale 11 peaks can be detected (2 peaks per day). For smaller scales of period 8h and 6h, the number of peaks observed are 17 and 22, respectively. The estimated mean time lag (“Lag”) between peaks was calculated.The bottom panel depicts the sum of these 4 relative intensity of the real wavelet transform coefficient (magenta) superimposed onto the corresponding actogram (black bars).

**SI Detrended fluctuation analysis**

Given a time series {Xt}, the Detrended Fluctuation Analysis (DFA) proposed by Peng et al. ^7^ and described in detail elsewhere^8, 9^, consists of five steps.In the first one, for each t ϵ {1,...N}, the cumulative walking time series Yt

$Y_{t}=\sum_{j=1}^{t} X_{j}$ (1)

is computed. This integrated time series {Y_t_} is divided into [N/n] non overlapping blocks, each containing *n* observations. In the third step, for each block, a least square line is fitted to the data, which represents the local trend of the block. In the fourth step, the time series {Y_t_} is detrended by computing

$Z_{t}=Y_{t}-Y_{t}^{n}$ (2)

where $Y_{t}^{n}$ denotes the adjusted fit on each block. Finally, in the fifth step, for each nϵ{2m+2,...,N/4}, the

$F(n)=\sqrt{\frac{1}{n}\sum_{t=1}^{M} (Y_{t}-Y_{t}^{n})^{2}}=\sqrt{\frac{1}{n}\sum_{t=1}^{M} Z_{t}^{2}}$ (3)

where M is the maximum multiple of *n*, smaller or equal to *N*, i.e. M=[N/n].Notice that F(n) will increase with block size *n*. A linear relationship on a log-log scale indicates the presence of power law scaling

$F(n)=\varphi n^{\alpha}$ (4)

Under such condition the fluctuations can be characterized by a scaling exponent α, which is the slope line when regressing log(F(n)) on log(n) where

1. 0 < α < 0.5 indicates intermediate memory
2. α = 0.5 indicates no correlations
3. 0.5 < α < 1 indicates long-term memory

By taking the logarithm of the root mean square fluctuation value given by step 4 we obtain

$$\log(F(n))=\log(\varphi)+\alpha\log(n) (5)$$

***Detrending***

The existence of trends (a smooth and monotonic or slowly oscillating pattern caused by external effects) in time series generated by biological systems is very common and almost unavoidable ^10^. Kantelhardt and colleagues (2001) reported that trends in the original time series data can lead to an artificial crossover in the slope of the log-log plot of F(*n*) vs. *n*, i.e., the slope α is increased for large time scales. To determine the order of DFA that would eliminate these trends, and to estimate the value of α reliably, DFA was calculated with different detrending orders^8^. For this purpose, linear (DFA1), square (DFA2), cubic (DFA3) and higher order polynomials were used in the fitting procedure. Since the detrending of *Y_j_*time series is performed by subtraction of the fits from data, these methods differ in their capability to eliminate data trends. In the n*^th^*order DFA (DFAn), trends in the profile of the *Y_j_* time series of order *m-1* are eliminated ^8^. In other words, the artificial crossover (more than one linear fit) disappears when the detrending order used in the DFA is larger than the order of the trend. An example of the detrending capacity of DFA is represented in Supplementary Figure 10 where crossovers are clearly observed for DFA1 and DFA2. Therefore, after evaluating the fit of all DFA performed with different detrending orders, we selected DFA3 as the lowest detrending order that eliminated trends in all data series and used it for comparingα-values between birds.


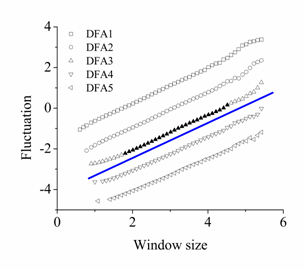


**Supplementary Figure 10:** Example of trend elimination capability of DFA in time series using linear (DFA1), quadratic (DFA2), cubic (DFA3) , 4th order (DFA4) and 5^th^ order (DFA5) DFA of the same time series analyzed in Fig. 7 of the main text. Note the presence of crossovers for large window sizes in DFA1 and DFA2 that is not observed for larger detrending orders. Therefore, DFA3 was selected for estimation of α-values given that 3rd order polynomials eliminated trends in data. Grey line represents the linear fit for DFA3 used for estimation.

***SI Scaling determination***

When using DFA, long-memory should not be a priori assumed; therefore, to reliably infer power-law scaling of the fluctuation function, a straight line in the log-log plot has to be established ^11^. Since a straight line is tantamount to a constant slope, the local slopes for a range of window sizes of log Fluctuation (F(n)) as a function of window size (*n*) have to be evaluated for constancy in an adequate range (Supplementary Fig. 6)^10, 11^. First, a straight line to log F (n) vs. log *n* within a small window is fitted. The window is then shifted successively over all calculated scales *n*. Panel A in Supplementary Figure 11 shows an example of how local slopes are obtained. In the example, 10 consecutive points in the double log plot of fluctuation *vs.* window size are fitted with a line, and the local slope, r^2^ and sum of squared residuals are estimated. Note that the fitting of the first 10 points (green triangles) showed a lower r^2^ and higher sum of squared residuals (see circled value in Supplementary Fig. 11C,D) compared to fitting data points in the middle range (blue triangles). The value of the local slope(Supplementary Fig.11B), r^2^ of the linear fit(Supplementary Fig. 11C) and the sum of squared residuals (Supplementary Fig. 11D) depend both on the location and number of data points utilized in the fitting procedure.


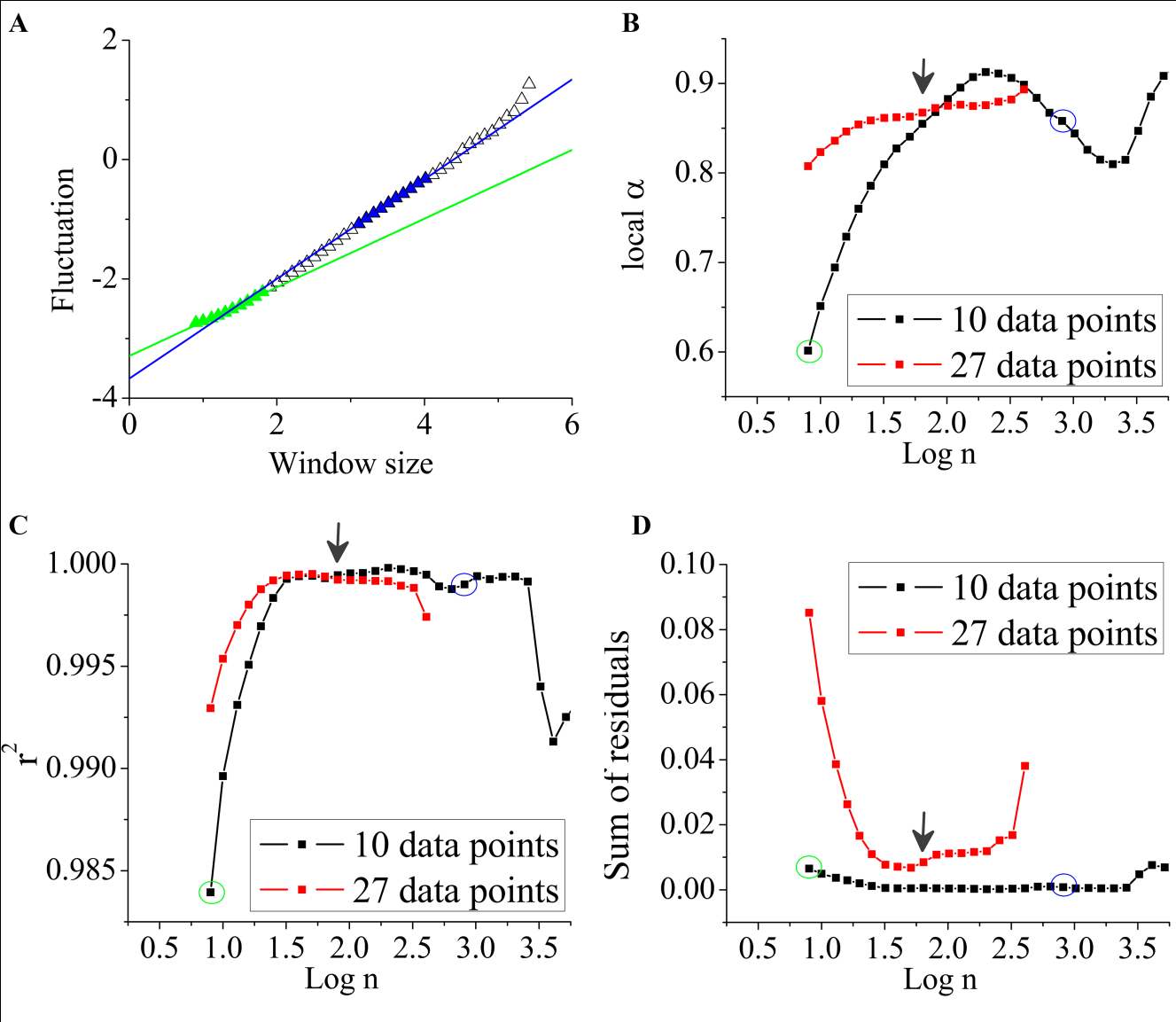


**Supplementary Figure 11: Example showing the criteria utilized for selecting the optimal region for α-estimation (grey arrow).** Third order DFA was performed on the same time series analyzed in Fig. 4. As depicted in panel A, local α-values were estimated form the slope of the linear fit for 10 data points (green or blue filled triangles), then the r^2^ and sum of square residuals of that fit were calculated. In panel B, the slopes estimated in panel A are circled in green or blue, respectively. This process was repeated over the entire data set for all possible length of data points and window size values. B) Local slopes, C) r^2^, and D) sum of square residuals of the corresponding linear fits estimated for different window sizes (10 and 27 data points). Note that bias in α estimation is low for 10 data points, showing greatest variability (B), lowest r^2^ (C), and higher sum of squared residuals (D) for both very small and large window sizes, indicating that the “middle” region is the optimal one. Complete analysis showed that the use of 27 data points from window sizes log(~30 s) and log(4.4 h) was optimal for α estimation in all animals (indicated by grey arrow and highlighted in blue in Fig. 7).

The appropriate scaling range was determined as the region for which all animals showed stable values of local slope, maximum coefficient of variation, and minimum sum of squared residuals ^9, 12^. This analysis showed that the use of 27 data points from window sizes log(~30 s) and log(4.4 h) was optimal for α estimation in all animals (indicated by grey arrow in Supplementary Fig.11 and highlighted in blue in Fig. 7). As a caveat, small window sizes produce lower α-values that trend towards values of 0.5, likely due to random spikes in the data set ^13^

As mentioned above, the presence of trends in the time series such as oscillations could potentially affect the correct estimation of α. Thus, the slow oscillatory trend contributed by the circadian rhythm should be eliminated by detrending through DFA. As a control of the detrending capacity of DFA, time series were filtered using a mean moving average with a period of 23.5 h to eliminate the underlying circadian rhythm and then reanalyzed with DFA and the results compared with those obtained from the analysis of the original, unfiltered, time series. Supplementary Fig.12A shows an example of the filtered time series compared to unfiltered data that, as expected, exhibits approximately the same α-value as the original unfiltered time series (Supplementary Fig. 12B), certifying that detrending reliably eliminated the effect of the circadian rhythm.


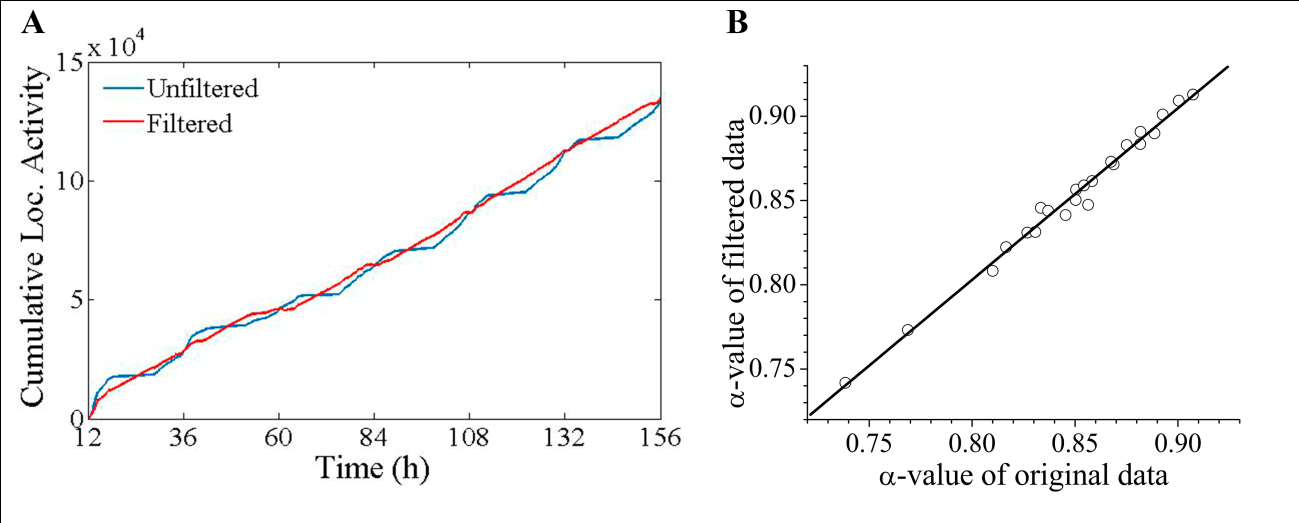


**Supplementary Figure12: Detrending capacity of DFA to eliminate the circadian rhythm.** A) Example of a time series (unfiltered) that was filtered using a moving average filter (filtered) in order to eliminate the circadian oscillation. When DFA was performed for window sizes between 30 s and 4.4 h, on both original unfiltered and filtered time series, the α-values obtained were very similar and highly correlated, y =1.02x-0.011 (r^2^ = 0.99).

**SI Frequency Distribution of the Duration of Immobility events (FDD-I)**

The FDD-I was determined for each animal by plotting the frequency *vs.* the duration of immobility events, using a double logarithmic scale. Achievement of a linear fit was considered as an indication of a power-law (fractal) distribution. In theory, for a linear fit the slope should be constant regardless of the number of data points used in its estimation. However, inclusion of data points from the tail of the power-law distribution could affect S_I_ estimation. Supplementary Fig. 13 illustrates how the number of event data points included in the estimation of S_I_ affects its value (Supplementary Figure 13).The estimation of S_I_was performed only for durations of events ≤ 250 s in all animals.


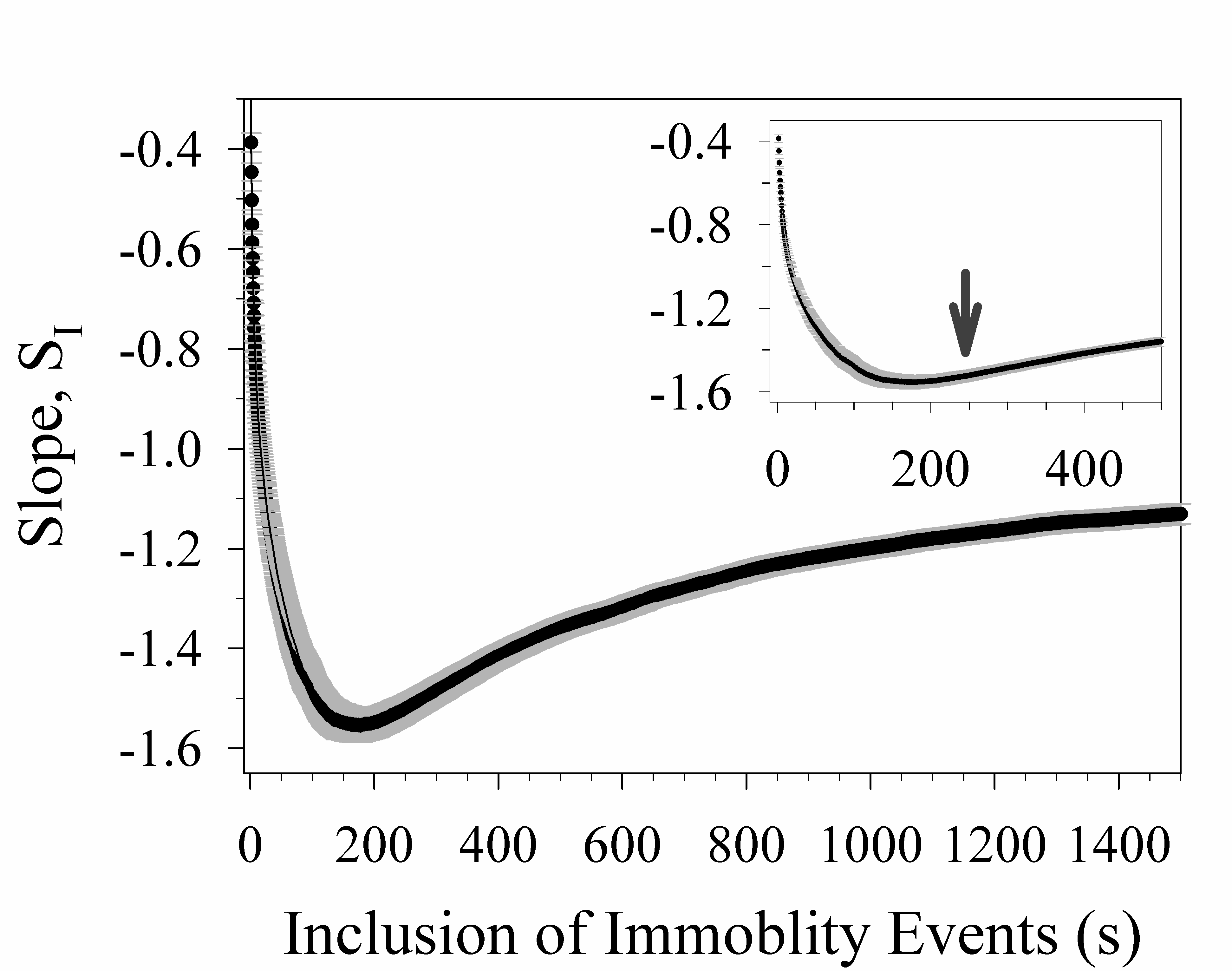


**Supplementary Figure 13: Example of criteria for selecting the optimal region for slope S_I_determination (black arrow)**

The slope (S_I_) in log-log frequency distribution of the duration of immobility events plots was estimated using an increasing number of immobility events duration starting from 1s. The inset is a zoom-in of the main figure. Notice that as the number of event data points included in the estimation of S_I_ increases, its value decreases to a minimum keeping a constant value thereafter for event sizes between 120 and 250 s (S_I_ ranges from -1.55 ± 0.03 to -1.52 ± 0.03). The inclusion of event data points above 250 s results in a subsequent increase in S_I_ values caused by long event durations in the tail of the power law distribution. Hence, for the estimation of S_I_ only durations of events ≤ 250 s were taken into account. The black line represents mean SI slope value and grey region the SEM values for all 24 birds.

Immobility events durations (i.e. an interval of time > 1s in which the animal remains immobile) were determined using the complete time series. The 0.5 s sampling rate is practically the resolution of a step, therefore the data of duration of immobility events was obtained with high resolution. Previous studies have shown that care should be taken when evaluating FDD from discretized behavior data, given that the bin size used can affect the slope of the distribution^18^. Therefore, the effect of using progressively larger bin sizes on the estimation of S_I_ was evaluated. Supplementary Figure 14 shows the FDD-I of 12 birds for bin sizes ranging from 0.5 to 60 s using durations of events ≤ 400 s for the estimation of S_I_. Of note is that for all bin sizes a power-law distribution can be observed, although the precise value of the slope is dependent upon bin size, showing a sharp increase for bin sizes larger than the mean immobility event duration (24 ± 2 s). Depending upon the definition of immobility events adopted herein, bin size limits the ability of detecting immobility events smaller than the bin size but has little effect on the estimation of large events.

**Supplementary Figure 14: Effect of bin size on frequency distribution of the duration of immobility events.** The original locomotor time series with 0.5s sampling rate recorded over 6.5 days (first panel), was binned (equivalent to reducing sampling rate) at increasingly large sizes as indicated in the title of each panel.The data from 12 birds (each a different color) are superimposed. The SI mean ± SEM slope for immobility event durations ≤ 400 s, is written in the upper right corner of each panel . Note that increasing bin size causes a decrease in SI especially for bins ≥ 30 s without changing the power law distribution. .

**SI Frequency distribution of the duration of mobility events (FDD-M)**

A mobility event was defined as an interval of time (> 1s) in which the animal remains mobile ^1, 9, 14, 15, 16, 17^. For each animal the FDD-M was analyzed by plotting the frequency *vs.* the duration of mobility events, using a double logarithmic scale. Quail locomotor time series showed many mobility events of long duration (Supplementary Fig. 15A), indicating an asymmetric frequency distribution of the duration of the immobility events; unlike the frequency distribution of the duration of immobility events, the duration of mobility events did not show such a clear power-law (fractal) distribution in the log-log plot as that shown in Supplementary Figure 11.


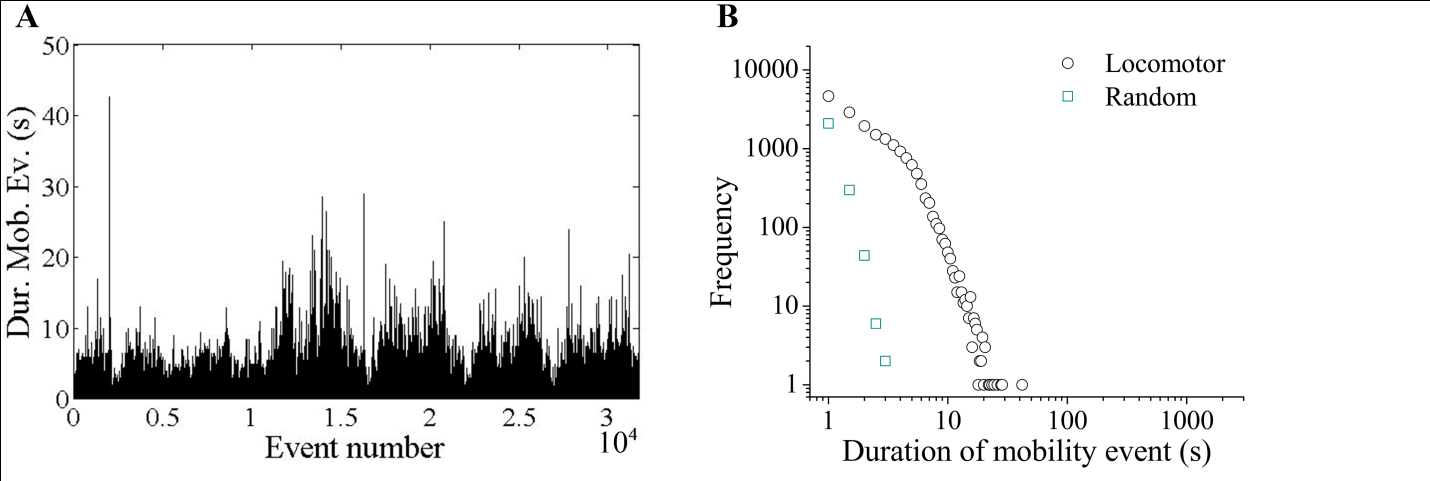


**Supplementary Figure 15: Frequency distribution of mobility events.** A) Mobility event duration as a function of the event number for one animal time series. B) Frequency distribution of the immobility events computed from the 6.5 days of continuous monitoring (black open circles). The same time series was randomized and then reanalyzed (blue open squares) for comparison.

In Supplementary Figure 16 we evaluated the effect of bin size on the FDD-M. Following the same rationale utilized for the 0.5 s sampling rate, if the bird showed any mobility during the time interval (bin) it was considered to have moved, even if the bird took only one step As bin size increases, the likelihood of longer mobility events also increases, thus causing a significant impact of bin size on the type of distribution. As aforementioned(Supplementary Fig. 15 and 16A), the duration of mobility events did not show a clear power-law (fractal) distribution in the log-log plot for the original 0.5 sampling rate bin, however for large bin sizes a power-law distribution became apparent.

**Supplementary Figure 16: Effect of bin size on frequency distribution of the duration of mobility events.** The original locomotor time series with 0.5s sampling rate recorded over 6.5 days (first panel), was binned (equivalent to reducing sampling rate) atincreasingly larger sizes as indicated from the title of each panel. The data from 12 birds (a different color each) are superimposed. Note that increasing bin size causes a change in the type of distribution, trending towards a power-law distribution for larger bin sizes.

**SI Power spectrum analysis provides additional evidence towards power-law long-range correlations in avian locomotor time series**

The power spectrum is widely used to measure correlations in a time series^32^. The fact that the log-log plot of the power spectrum *S*(ƒ) vs ƒ is linear implies, *S*(ƒ) ~ ƒ^-β^. The exponent β is related to the mean fluctuation exponent α by β = 2α - 1 and can serve as an indicator of the presence and type of correlations^19^. Supplementary Figure 17 shows that the log-log plot of the power spectrum is linear, with a slope ***-***1.025 ± 0.007, providing additional evidence towards power-law long-range correlations in avian locomotor time series

**Supplementary Figure 17: Log-log plot of Power Spectrum Analysis,**  The same power spectrum represented in Fig. 2A is plotted on a double logarithmic scale. The estimated slop for all data points (β) is approximately 1, indicating long-range correlations in locomotor time series.

**References**

1. Kembro, J. M., Guzman, D. A., Perillo, M. A. & Marin, R. H. Temporal pattern of locomotor activity recuperation after administration of propofol in Japanese quail (Coturnix coturnix japonica). *Res Vet Sci***93**, 156-162 (2012).

2. Guinebretiere, M., Huneau-Salaun, A., Huonnic, D. & Michel, V. Plumage condition, body weight, mortality, and zootechnical performances: the effects of linings and litter provision in furnished cages for laying hens. *Poult Sci***92**, 51-59 (2013).

3. Ghosh, S., Manimaran, P. & Panigrahi, P. K. Characterizing multi-scale self-similar behavior and non-statistical properties of fluctuations in financial time series. *Phys A***390**, 4304-4316 (2011).

4. Leise, T. L. & Harrington, M. E. Wavelet-based time series analysis of circadian rhythms. *J Biol Rhythms***26**, 454-463 (2011).

5. Pering, T. D., Tamburello, G., McGonigle, A. J. S., Hanna, E. & Aiuppa, A. Correlation of oscillatory behaviour in Matlab using wavelets. *Comput Geosci***70**, 206-212 (2014).

6. Farge, M. Wavelet transforms and their applications to turbulance. *Annu Rev Fluid Mech***24**, 395-457 (1992).

7. Peng, C. K., Buldyrev, S. V., Havlin, S., Simons, M., Stanley, H. E. & Goldberger, A. L. Mosaic organization of DNA nucleotides. *Phys Rev E***49**, 1685-1689 (1994).

8. Kantelhard, J. W., Koscielny-Bunde, E., Rego, H. H. A., Havlin, S. & Bunde, A. Detecting long-range correlations with detrended fluctuation analysis. *Phys A***295**, 441-454 (2001).

9. Kembro, J. M., Flesia, A. G., Gleiser, R. M., Perillo, M. A. & Marin, R. H. Assessment of long-range correlation in animal behavior time series: The temporal pattern of locomotor activity of Japanese quail (Coturnix coturnix) and mosquito larva (Culex quinquefasciatus). *Phys A***392**, 6400-6413 (2013).

10. Hu, K., Ivanov, P. C., Chen, Z., Carpena, P. & Stanley, H. E. Effect of trends on detrended fluctuation analysis. *Phys Rev E***64**, 011114 (011119 pages) (2001).

11. Maraun, D., Rust, H. W. & Timmer, J. Tempting long-memory – on the interpretation of DFA results. *Nonlinear Proc Geoph***11**, 495-503 (2004).

12. Macintosh, A. J., Pelletier, L., Chiaradia, A., Kato, A. & Ropert-Coudert, Y. Temporal fractals in seabird foraging behaviour: diving through the scales of time. *Sci Rep***3**, 1884 (2013).

13. Chen, Z., Ivanov, P. C., Hu, K. & Stanley, E. Effects of nonstationaries on detrended fluctuation analysis. *Physical Review E***65**, 041107 (041115 pages) (2002).

14. Kembro, J. M., Perillo, M. A., Pury, P., Satterlee, D. G. & Marin, R. H. Fractal analysis of the ambulation pattern of Japanese quail. *Br Poult Sci***50**, 161–170 (2009).

15. Kembro, J. M., Satterlee, D. G., Schmidt, J. B., Perillo, M. A. & Marin, R. H. Open-field temporal pattern of ambulation in Japanese quail genetically selected for contrasting adrenocortical responsiveness to brief manual restraint. *Poult Sci***87**, 2186-2195 (2008).

16. María, G. A., Escós, J. & Alados, C. L. Complexity of behavioural sequences and their relation to stress conditions in chickens: a non-invasive technique to evaluate animal welfare. *Appl Anim Behav Sci***86**, 93-104 (2004).

17. Rutherford, K. M., Haskell, M., Glasbey, C., Jones, R. B. & Lawrence, A. Detrended fluctuation analysis of behavioural responses to mild acute stressors in domestic hens. *Appl Anim Behav Sci***83**, 125-139 (2003).

18. Lo, C. C.*, et al.* Common scale-invariant patterns of sleep-wake transitions across mammalian species. *Proc. Natl. Acad. Sci. U. S. A.***101**, 17545-17548 (2004).

19. Ivanov, P. C.*, et al.* From 1/f noise to multifractal cascades in heartbeat dynamics. *Chaos***11**, 641-652 (2001).
